# Eco‐evolutionary dynamics of Atlantic cod spatial behavior maintained after the implementation of a marine reserve

**DOI:** 10.1111/eva.13483

**Published:** 2022-10-09

**Authors:** David Villegas‐Ríos, Carla Freitas, Even Moland, Esben M. Olsen

**Affiliations:** ^1^ Instituto Mediterráneo de Estudios Avanzados (CSIC‐UiB) Esporles Spain; ^2^ Instituto de Investigaciones Marinas (IIM‐CSIC) Vigo Spain; ^3^ Institute of Marine Research His Norway; ^4^ MARE, Marine and Environmental Sciences Center Madeira Tecnopolo Funchal Portugal; ^5^ Department of Natural Sciences, Centre for Coastal Research (CCR) University of Agder Kristiansand Norway

**Keywords:** conservation ecology, fish behavior, fully‐protected area, individual behavior, marine protected areas, movement ecology, survival

## Abstract

The effects of marine reserves on the life history and demography of the protected populations are well‐established, typically increasing population density and body size. However, little is known about how marine reserves may alter the behavior of the populations that are the target of protection. In theory, marine reserves can relax selection on spatial behavioral phenotypes that were previously targeted by the fishery and also drive selection in favor of less mobile individuals. In this study, we used acoustic telemetry to monitor the individual spatial behavior of 566 Atlantic cod (*Gadus morhua* Linnaeus, 1758) moving within a marine reserve and a control site in southern Norway, starting 1 year before the implementation of the marine reserve and lasting up to 9 years after. Following a *before‐after‐control‐impact* approach, we investigated changes in (1) survival, (2) selection acting on behavioral traits, and (3) mean behavioral phenotypes, after the implementation of the marine reserve. We focused on three behavioral traits commonly used to describe the mobility of aquatic animals: home range size, depth position, and diel vertical migration range. Survival increased after reserve implementation, but contrary to our expectations, it subsequently decreased to preprotection levels after just 3 years. Further, we found no significance in selection patterns acting on any of the three behavioral traits after reserve implementation. Although some changes related to water column use (the tendency to occupy deeper waters) were observed in the marine reserve after 9 years, they cannot unequivocally be attributed to protection. Our results show that survival and behavioral responses to marine reserves in some cases may be more complex than previously anticipated and highlight the need for appropriately scaled management experiments and more integrated approaches to understand the effects of marine protected areas on harvested aquatic species.

## 1 INTRODUCTION

For decades, science on marine protected areas (MPAs) has focused on the demographic effects of protection and the benefit to fisheries. Protection typically increases survival, mean size, and age within the protected populations resulting in a *filling‐in* of demographic structures (Fernández‐Chacón et al., 2020; Moland et al., 2013, 2021; Taylor & McIlwain, 2010). Such demographic changes are in turn expected to benefit fisheries beyond MPA boundaries through the net export of pelagic eggs and larvae (the recruitment effect) and spillover of postsettled juveniles and mature fish (Abesamis & Russ, 2005; Di Lorenzo et al., 2016).

Although not often considered, protection within MPAs may also conserve behavioral phenotypes that are otherwise often altered by human exploitation (Bergseth et al., 2016). For instance, several studies have shown how the exclusion of extractive activities in MPAs can lead to bolder and more naïve fish populations (Bergseth et al., 2016; Januchowski‐Hartley et al., 2015). However, the ability of MPAs to restore other aspects of the behavior of the target populations, such as movement traits, remains untapped. This is a critical gap in conservation science because movement behavior is crucial to understand MPA effectiveness (Thorbjørnsen et al., 2021; Villegas‐Ríos et al., 2021). Moreover, changes in movement‐related traits, for instance those resulting from MPA protection, can impact key aspects of population dynamics (e.g., survival, growth, and dispersal) and patterns of connectivity, thus affecting the resilience and recovery potential of populations (Anthony & Blumstein, 2000; Arlinghaus et al., 2017; Wong & Candolin, 2015).

Short‐term changes in movement behavior inside MPAs may take place through relaxed selection against movement phenotypes that otherwise, i.e., in fished areas, may regulate vulnerability to fishing (Diaz Pauli & Sih, 2017). For instance, Olsen et al. (2012) reported that trapping, angling, and gillnetting selected against Atlantic cod individuals (*Gadus morhua* Linnaeus, 1758) that displayed extensive diel vertical migrations and used shallower waters (where fishing activities are concentrated), and against individuals with a higher tendency to display linear horizontal movements. Quinn et al. (2007) showed that angling and gillnetting are selectively favoring early migration in salmon (*Oncorhynchus nerka* (Walbaum, 1792)). Last, when catchability depends on encounter between fish and fishers, selection may favor individuals with little activity (Alós et al., 2012; Biro & Post, 2008; Villegas‐Ríos et al., 2014). It is therefore expected that, once fishing is removed, selection acting on these and other movement traits is buffered or eliminated. Alternatively, short‐term changes in spatial behavior after protection may result from adaptive responses to changes in density and competition inside the MPA (Baskett & Barnett, 2015) or as an adaptation to reduced disturbance from fishing gears (Strain et al., 2019).

Marine protected areas may also initiate selection on behavioral traits that were not previously selected by the fishery. For instance, individuals with larger home ranges or dispersal ability of the protected populations may experience reduced fitness by spending more time at risk, i.e., outside the MPA, as compared to fish with smaller home ranges (Baskett & Barnett, 2015; Parsons et al., 2010; Villegas‐Ríos et al., 2021; Villegas‐Ríos, Moland, et al., 2017). This is expected to be particularly important under situations when intensive fishing takes place right at the border of the MPA (e.g., Kellner et al., 2007) and when the MPA fails to protect a significant part of the range of movements of animals present in the target population.

Short‐term changes in behavior due to altered selection patterns can be expected to be maintained in the long‐term through evolutionary processes as long as behavioral traits are heritable and not swamped by gene flow or via correlated selection on other traits (Baskett et al., 2007; Baskett & Barnett, 2015; Villegas‐Ríos, Moland, et al., 2017). Although there is little evidence on the heritability of fish behavioral traits relevant to conservation, a recent meta‐analysis reported an average heritability of 0.24 for animal behavioral traits, with behavioral traits related to dispersal and migration reaching 0.46 (Dochtermann et al., 2019).

To date, virtually all empirical studies on the protection effects of behavior focused on a single axis, wariness‐naïveté (Bergseth et al., 2016; Januchowski‐Hartley et al., 2015), and none compared pre‐ and postprotection scenarios using a before‐after‐control‐impact (BACI) design. This is not surprising as it requires continuous tracking of at least two populations (control and impact) before and after protection. Here we fill this science gap by focusing on the movement behavior of Atlantic cod in southern Norway for over a decade. We monitored individual behavior of 566 acoustically tagged Atlantic cod in an MPA and a control site, starting 1 year before protection and lasting up to 9 years after MPA implementation. We focused on three behavioral traits that are typically used to describe the spatial behavior of fish, and that had been previously identified as potential fitness determinants: home range size, depth position, and diel vertical migration. We hypothesized that (1) survival would increase in the MPA site after the implementation of protection measures and that (2) relaxing fishing mortality inside the MPA would alter the selection of cod behavioral traits. We further hypothesized that (3) changes in behavioral selection patterns would change the mean behavioral phenotypes in the protected population over time.

## 2 MATERIAL AND METHODS

### 2.1 Study species

The Atlantic cod is a large‐bodied and long‐lived fish, with a maximum length of more than 140 cm and maximum age of more than 20 years (Hutchings & Myers, 1993; Kenchington & Kenchington, 1993), for which healthy populations have key functions in North Atlantic coastal ecosystems (Frank et al., 2011; Norderhaug et al., 2021). However, the species is also a valued target for commercial and recreational fishers and has suffered severe depletions throughout much of its range. Coastal Skagerrak hosts at least two cod ecotypes. On the one hand, genetically distinct local populations exist on a fjord scale, probably maintained by a combination of restricted movement and spawning in areas sheltered from the prevailing coastal current (Ciannelli et al., 2010; Knutsen et al., 2011). Fjord cod typically complete their life cycle inside the fjords, including foraging and spawning activities, which take place between February and April (Knutsen et al., 2007). On the other hand, there are cod that are genetically similar to oceanic cod from the North Sea (Knutsen et al., 2018). Cod in the North Sea and Skagerrak have suffered a near‐collapse in recent decades, likely linked to a combined negative impact of intense exploitation and climate change (Beaugrand et al., 2003; Olsen et al., 2011; Rogers et al., 2011). In coastal Skagerrak, recreational fishing with hook and line is the single most important component of fishing mortality, followed by commercial fishing with fixed gears such as traps and nets, and thereafter recreational fishing with similar fixed gears (Kleiven et al., 2016). The total fishing pressure on the coastal populations is considered unsustainable, with annual survival rates typically in the range of 0.2–0.3 only (Fernández‐Chacón et al., 2017). The proportion of deaths due to fishing (as opposed to natural causes) is typically above 0.6 and for larger mature cod it approaches 1 (Fernández Chacón et al., 2015). Cod fisheries in coastal Skaerrak are therefore typically size‐selective (see also Olsen & Moland, 2011). Fishing peaks during the summer months of June–August, coinciding with the main holiday season in Norway (Kleiven et al., 2016).

### 2.2 Study area and telemetry arrays

**FIGURE 1:**
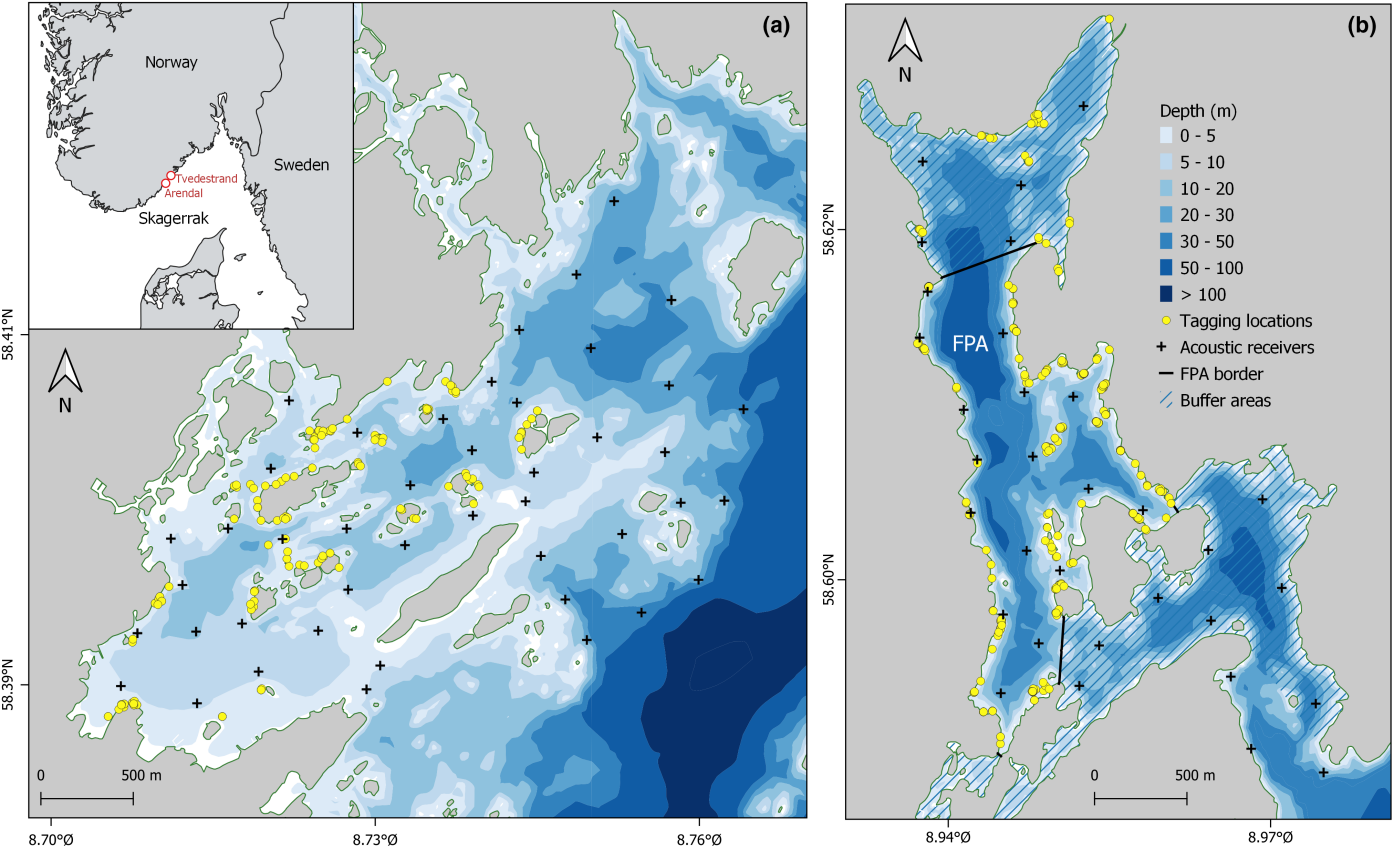
Map of the study area and telemetry arrays. The study was conducted in the south of Norway at two sites connected to the Skagerrak Sea: (a) the Sømskilen area, in Arendal municipality, that served as control and (b) the Tvedestrand fjord in Tvedestrand municipality where a marine protected area that includes a fully‐protected area (FPA), and two buffer areas was implemented in 2012. Telemetry arrays consisted of 44 receiver stations in Sømskilen and 32 in Tvedestrand.

Our study was carried out in two different sites separated by ~30 km on the southern Norwegian coast. To the south, the Sømskilen site (hereafter the control area) is a semi‐sheltered embayment that includes numerous islands and islets and has a maximum depth of about 40 m along the eastern limit of the receiver array (Figure 1). To the north, the Tvedestrand site (hereafter the MPA) is a fjord with several sills and basins, extending 8 km inland from the open ocean (Figure 1). The Tvedestrand fjord is shallower in its outer southern parts, while the inner northern sector is deeper (up to 90 m, Figure 1). The MPA included a fully‐protected area (FPA) effectuated in June 2012 of ~150 ha, where all types of fishing are forbidden. This FPA is surrounded by a buffer zone where angling is allowed but commercial fishing gears such as trammel nets are banned. The MPA was set up primarily to protect cod around a known spawning locality with associated nurseries. Two Innovasea VR2W telemetry arrays (44 receivers in the control area covering a surface of ~6 km^2^, and 32 receivers in the MPA covering ~4 km^2^) were deployed to monitor fish behavior (Figure 1). Cod behavior was recorded during more than nine consecutive years in Tvedestrand (June 2011–December 2020) and four consecutive years in Sømskilen (June 2011–May 2014). All receivers were deployed at about 3 m depth with the hydrophone pointing down, attached to moorings, and held buoyant with subsurface trawl balls. Overall, receivers were positioned to ensure comprehensive monitoring of the study areas (Wiig et al., 2013). Range testing in both the control area and the MPA suggested very good coverage and the ability to track fish uninterruptedly as long they were moving inside the telemetry arrays (Freitas et al., 2015, 2016; Moland et al., 2019; Wiig et al., 2013). Fish detection data, consisting of records of tag identity, tag depth, tag detection time, and receiver identity, were downloaded twice per year from the receivers while maintenance of the array was conducted once per year.

### 2.3 Capture and tagging

A total of 566 cod were captured and tagged (control area: *n* = 156, MPA: *n* = 410). Within the MPA, the majority of fish were tagged inside the FPA, except in 2019 and 2020 when a total of 24 and 10 fish, respectively, were tagged in the northern buffer area (Figure 1b). Fish were captured using fyke nets left soaked for 1–3 days at depths ranging from 1 to 10 m. Cod selected for tagging were anaesthetized in clove oil at 40 mg/L (Munday & Wilson, 1997) and equipped with Innovasea V9P or V13P transmitters inserted in the abdominal cavity (see Olsen et al., 2012). For that, a cut of about 2 cm was made in the abdomen of the fish and afterward closed with two stitches using absorbable surgical thread. Previous experiments showed negligible tagging mortality (Freitas et al., 2015; Olsen et al., 2012; Villegas‐Ríos, Réale, et al., 2017). Transmitters provide information on the current depth along with an identity code for each tag. In 2011 and 2012 the maximum depth registered by the transmitters was 100 m (0.44 m resolution and 5 m accuracy) and in 2013–2019 the maximum depth was 50 m (0.22 m resolution and 2.5 m accuracy). Transmitters were set to transmit a signal every 110–250 s, with a random interval to reduce code collision, and with an expected battery life between 508 and 1292 days, depending on the transmitter type. Following full recovery from anesthesia (typically 5–10 min) all cod were released at their capture location.

### 2.4 Estimation of behavioral traits and fate

For each tagged cod, centers of activity (COA) were calculated for every 30 min time bin (Simpfendorfer et al., 2002). Time series of depth position, COA latitude, and COA longitude were plotted and used to identify and remove all detections recorded after cessation of movement had occurred, that is, when the fish appeared to be dead (Villegas‐Ríos et al., 2020). Code collisions and false detections were eliminated by using a minimum of two detections per 24 h period filter (Villegas‐Ríos et al., 2013).

For each individual, we estimated three behavioral traits: home range size (on a weekly basis), diel vertical migration (on a daily basis), and the mean depth position (on a daily basis). Home range size for each week was estimated as the kernel utilization distribution with a probability level of 95% using all the COAs from that week, using the library *adehabitatHR* (smoothing factor = 50, extent = 2) (Calenge, 2006). Weekly estimates of the home range were only computed when fish were present in the array during at least 4 days in a particular week (not necessarily consecutive). Diel vertical migration range was estimated as the difference between average depth during daytime and average depth during nighttime (Freitas et al., 2015). Mean depth position was calculated as the average depth for each day (24 h period).

Fate of each individual was assigned based on Villegas‐Ríos et al. (2020). In brief, time series of depth, COA latitude, and COA longitude were used to classify the fish as either: (1) survived within the study area (i.e., multiple detections indicated horizontal and vertical movements until the end of the battery life of the transmitter), (2) dispersed from the study area (i.e., detections indicated directional movement toward the outermost receivers followed by an absence of detections for the rest of the study), (3) natural mortality when the fish stopped showing horizontal and vertical activity (usually with continued signals from a fixed depth within the study area) or (4) harvested within the study area when the fish disappeared from the receiver array before the end of the battery life and the last detections came from receivers not on the edge of the array. In this study, since we were interested in the overall patterns of selection acting on the local cod populations, natural and harvest mortality were pooled, so our mortality computes all cases of death. Fate could not be assigned to 22 fish either because they had no enough data to accurately assign a fate, or fate was doubtful so a total of 544 fish were considered for survival analyses.

### 2.5 Survival analysis

Survival models were run separately for the control area and the MPA using the *survfit* function in the (Therneau, 2022) *survival* library in R. Fate (dead = 1, alive = 0) and fate date for each fish were used as response variables. Note that survival models specifically model “time to event” (in this case mortality) and therefore account for the differences in observation periods (i.e., tracking dates) of our individuals. To simplify the analyses, the different cohorts of tagged cod were grouped into four different “periods”: “before” grouped fish tagged in the year 2011, i.e., before MPA implementation, “after1” included fish tagged in 2012 and 2013 and corresponds to the period of time for which data from both the control area and the MPA was available. Two extra groups were defined in the MPA: “after2” for fish tagged in 2014–2016, and “after3” for fish tagged in 2017–2020. Period was the only explanatory variable included in the survival models. Kaplan–Meyer curves were constructed for each study area and period, and differences between periods within each study area were assessed using log‐rank tests. Fish that dispersed from the telemetry arrays were considered to be alive (i.e., survivors) until the dispersal date in the survival models. Note that survival and dispersal are considered separately as two different fates in our fish, that can be unequivocally identified from the patterns of detections, as explained in more detail in Villegas‐Ríos et al. (2020).

### 2.6 Selection on behavioral traits

To formally investigate changes in selection acting on behavioral traits after MPA implementation, we run a binomial model using the probability of surviving the first year (*S*) after tagging date as the response variable (1 = “survived,” 0 = “died”):

$$\begin{array}{cc} S= & \;\alpha +\beta_{1}\text{Depth}+\beta_{2}\mathrm{DVM}+\beta_{3}\mathrm{HR}+\beta_{4}\text{Body size}\\ & \;+\beta_{5}\text{Site\_period}+\beta_{6}\text{Depth}*\text{Site\_period}\\ & \;+\beta_{7}\mathrm{DVM}*\text{Site\_period}+\beta_{8}\mathrm{HR}*\text{Site\_period}\\ & \;+\beta_{9}\text{Body}\;\text{size}*\text{Site\_period}+\varepsilon_{i,t} \end{array}$$

The model included data from 2011–2013 (i.e., the before period and after1 period) when both the MPA and the control area were monitored. The following explanatory variables were included: mean depth position per day (*Depth*), diel vertical migration (*DVM*), home range size (*HR*), *Body size* (fish length when tagged), and *Site_period* (four levels: “control before,” “control after1,” “MPA before,” MPA after1”). As behavioral traits were estimated several times per fish, we used the mean values from June, the month following the tagging season, as a common descriptor of the behavior of each fish, under the assumption that individual behavioral traits are repeatable over time (Villegas‐Ríos, Réale, et al., 2017). All individuals that had dispersed or died before the end of June were removed from the analyses. We ended up with a dataset of 252 individuals (137 in the control area and 115 in the MPA). Then, we included the interaction between *Site_period* and the different behavioral traits and body sizes in the model. In order to test for changes in selection after protection, we performed multiple post hoc pairwise comparisons on the full model, prior to model selection. More specifically, we tested whether any slope (*β* coefficients) was different from zero and whether differences in slopes between periods (“before” vs. “after1”) on each site (control and MPA) were significant using the *testInteractions* function in the *phia* library (De Rosario‐Martinez et al., 2015), and the *p*‐value adjustment method of Benjamini and Hochberg (1995). Then, model selection was performed by using the *setpAIC* function in R (Johnson & Omland, 2004).

In a separate model, we further investigated changes in selection in the MPA using all available data from the MPA (2011–2020) for which behavior in June could be calculated (*n* = 320 individual cod). This model included the same response and explanatory variables as explained above, but they interacted with the variable *period* (instead of *Site_period*) that in this case included the four different levels: “before”, “after1”, “after2”, and “after3”.

Given that results of the logistic models could be impacted by the time frame considered to classify fish as either dead or alive (i.e., the period of selection, which was 1 year in our models above), we ran similar models considering alternative periods of selection from 60 to 500 days reaching the same conclusions (results not shown).

### 2.7 Change of spatial behavioral traits over time

To formally explore changes in the mean population behavioral phenotypes after protection, we ran three mixed‐effects additive models (GAMMs) using each of the three behavioral traits as response variables:

$$\begin{array}{cc} {\text{Behaviour}}_{i,t}= & \;\alpha +\beta_{1}{\text{period}}_{t}+\beta_{2}{\text{site}}_{i}+\beta_{3}{\text{period}}_{t}*{\text{site}}_{i}\\ & +f_{1}\left(\text{time}\right)*{\text{site}}_{i}+f_{2}\left({\text{Body size}}_{i}\;\right)*{\text{site}}_{i}\\ & +f_{3}\left(\mathrm{ID}\right)+\varepsilon_{i,t}, \end{array}$$

where ${\text{Behaviour}}_{i,t}$ represents depth position (square root transformed), diel vertical migration, or home range size (log‐transformed) of individual *i* on time *t*. These models included data from both the control area and the MPA and used data from the “before” and the “after1” periods (2011–2014). The models included period (“before,” “after1”), site (“control area,” “MPA”) and their interaction as explanatory variables. A significant interaction between period and site would mean that behavior changed in a different direction in the control area and the MPA. Body size and time, and their interaction with the site were also included as explanatory variables to account for body size effects and seasonal changes in behavior. For that, we used nonparametric smoothing functions (*f*_*n*_) with thin plate splines, fitted with four knots in order to avoid overfitting. Time corresponds to the day of the year (from 1 to 365) in the depth and diel vertical migration models, and week (from 0 to 52) in the home range size model. We further included individual identity as a random‐effect smoother and temporal autocorrelation term to account for the structure of the data. Model selection was not performed as we were interested in testing the effect of all the explanatory variables according to our hypothesis and we wanted to compare the effects of the different models (Sarmento & Berger, 2017). Models were fitted using the *bam* function in library *mgcv* in R (Wood, 2017).

To further investigate changes in behavior over time in the MPA, we run an extra set of mixed models (one per behavioral trait) including all data available in the MPA (2011–2020) with a similar model structure as above but excluding site and instead including the four periods (covariate ${\text{period}}_{t}$):

$${\text{Behaviour}}_{i,t}=\alpha +\beta_{1}{\text{period}}_{t}+f_{1}\left(\text{time}\right)+f_{2}\left({\text{Body size}}_{i}\;\right)+f_{3}\left(\mathrm{ID}\right)+\varepsilon_{i,t}$$

Note that in all the models in this section, the variable period was defined in a slightly different way as compared to the survival and selection models (previous sections). Here, “before” grouped behavioral traits estimated for the period May 2011–June 2012, i.e., behavioral traits before MPA implementation; “after 1” included data from July 2012 to December 2014 and corresponds to the period of time after MPA creation for which data from both the control area and the MPA was available. Two extra groups were defined in the MPA: “after2” for data from the period January 2015 to December 2017 and “after3” for data from the period January 2018 to December 2020, i.e., the end of the study period. Equivalent models were run using “years” instead of “periods” yielding similar results (not shown).

## 3 RESULTS

The size of the fish tagged in the control area (range: 30–80 cm; mean = 46.1 cm) and the fish tagged in the MPA (range: 30–75 cm; mean = 46.6 cm) was similar (*t*‐test with *t* = −0.58; *p* = 0.561, Figure S1). More than 60 million detections were downloaded from the two receiver arrays in more than 9 years of duration of this study. After quality control and filtering, we obtained 34,551,055 depth data points and estimated a total of 5,518,340 COAs. The presence of fish in the arrays averaged 31 weeks, but there was a great variation with some fish having data for just 1 week and others for 129 weeks (Figure S2). Most of the fish were resident in the study areas (i.e., they were present in the telemetry arrays as long as they were alive) with only 25% of the individuals dispersing from the control site and 10% from the MPA (considering both the FPA and the buffer areas) during the study period. Behavioral traits varied within and between study areas over the years (Table 1). Weekly home range size in the control area ranged between 0.04 and 1.50 km^2^, whereas it ranged between 0.04 and 1.66 km^2^ in the MPA. Diel vertical migration varied between −16.7 and 28.2 m in the control area and between −30.4 and 31.6 m in the MPA. Mean depth position ranged between 0 and 56.1 m in the control area and between 0 and 55.2 m in the MPA (Figure S3).

**TABLE 1:** Summary of the tagged individuals and mean (minimum–maximum) behavioral traits for each cohort of tagged cod

Site	Year	Number of individuals tagged	Tracking time (days)	Body size (cm)	Depth position (m)	Diel vertical migration (m)	Home range size (km^2^)
Control	2011	51	203 (10–510)	45.5 (31–69)	9.66 (0–53.1)	2.05 (−15.2 to 24.1)	0.182 (0.041–0.788)
	2012	80	202 (1–526)	47.1 (30–80)	12.6 (0.811–56.1)	3.90 (−16.7 to 28.2)	0.254 (0.041–1.5)
	2013	25	194 (40–395)	43.7 (31–64)	15.0 (0–55.2)	2.58 (−14.5 to 22.7)	0.249 (0.043–1.26)
MPA	2011	51	322 (6–1452)	46.3 (30–67)	14.1 (0.399–54.4)	2.77 (−30.4 to 28.7)	0.134 (0.046–0.936)
	2012	70	331 (9–909)	46.7 (30–75)	14.7 (1.3–53.8)	2.87 (−27.3 to 31.6)	0.168 (0.039–1.66)
	2013	25	301 (6–519)	45.3 (30–64)	15.6 (1.17–44.8)	2.34 (−25.1 to 21.8)	0.169 (0.040–1.4)
	2014	65	266 (11–792)	43.8 (30–50)	15.4 (0–55.2)	3.57 (−26.8 to 27.7)	0.165 (0.042–1.45)
	2015	30	216 (7–885)	50.8 (35–68)	14.9 (0.349–53.7)	3.93 (−21.1 to 24.7)	0.135 (0.040–1)
	2016	25	305 (38–932)	50.1 (34–74)	15.4 (0.755–46.1)	2.11 (−18.5 to 28.7)	0.119 (0.046–0.815)
	2017	25	373 (98–835)	45.6 (35–61)	15.9 (0.0733–51.6)	2.69 (−25.8 to 30.8)	0.145 (0.041–1.13)
	2018	27	218 (16–624)	48.2 (37–60)	12.6 (0.152–44.9)	1.61 (−30.4 to 21.1)	0.169 (0.049–0.996)
	2019	62	249 (0–525)	47.2 (30–72)	13.8 (0–44.6)	1.96 (−22.9 to 19.2)	0.141 (0.041–0.955)
	2020	30	149 (36–211)	46.6 (32–67)	11.2 (0.471–26.8)	2.83 (−14.2 to 20.2)	0.132 (0.048–0.77)

### 3.1 Survival

**FIGURE 2:**
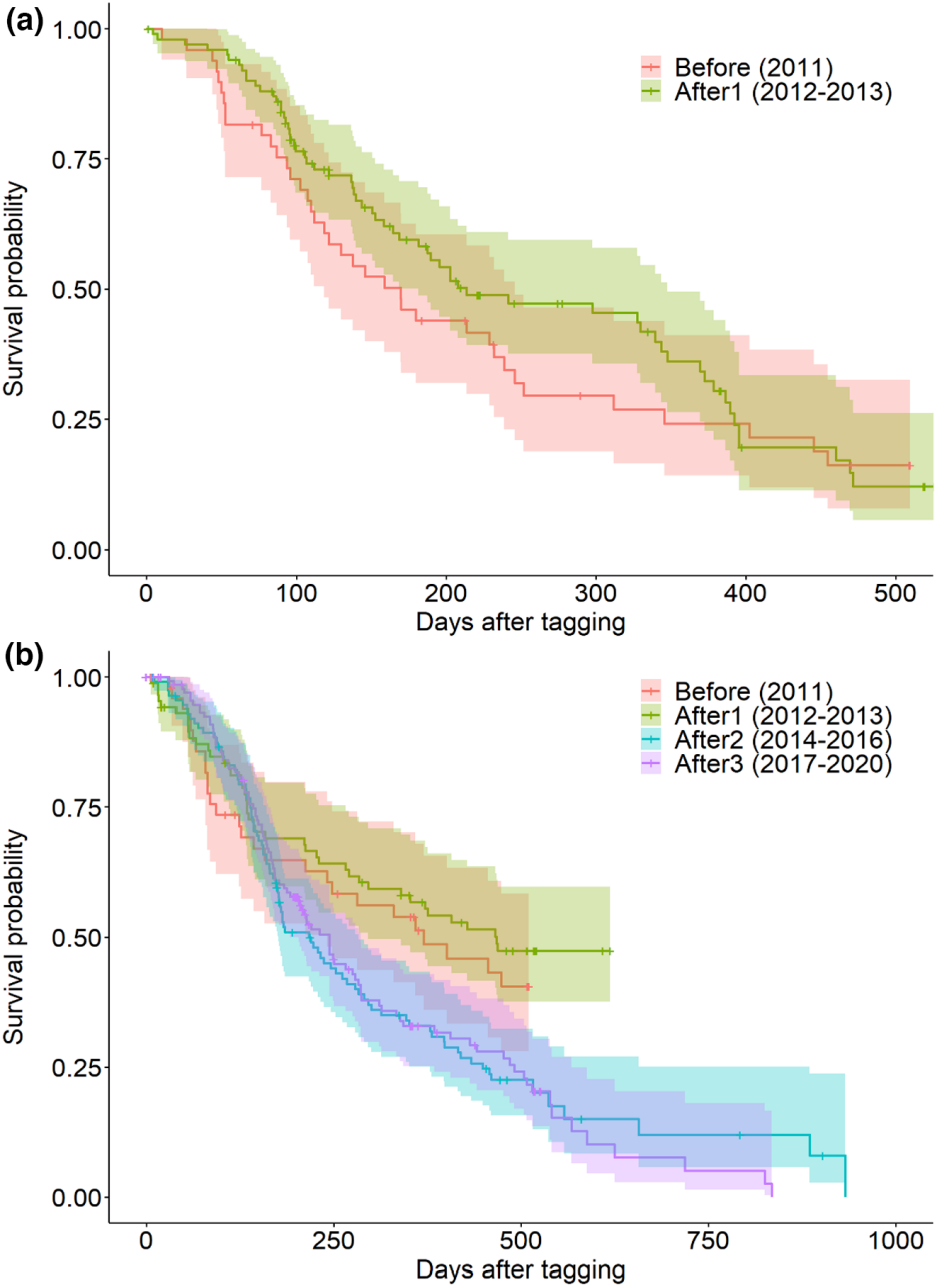
Kaplan–Meyer curves of the survival probability for Atlantic cod tagged in different periods (years in brackets) in (a) the control area, where no significant differences between periods were detected, and (b) the MPA where survival curves differed significantly (see main text). Note the different limits in the *X*‐axis between both plots.

Survival was, in general, low: in all areas and periods, the probability of surviving 1 year was lower than 0.57, as shown by the survival curves (Figure 2). The survival probability in the control area did not differ significantly between the two periods analyzed (log‐rank test with *p*‐value = 0.36; *df* = 1; *χ* ^2^ = 0.8) (Figure 2a). The probability of surviving the first year after tagging (P_1year_) in the control area was 0.24 (CI: 0.14–0.41) with a median survival time (*M*_s_; the time at which 50% of the population has died) of 169.3 days (CI: 118.4–245.5), for fish tagged in the “before” period. For fish tagged in the “after1” period, P_1year_ was 0.36 (0.26–0.49) and *M*_s_ was 213.5 (181.0–347.5).

In the MPA there was an overall effect of period on the survival probability (log‐rank test with *p*‐value < 0.002; *df* = 3; *χ* ^2^ = 15.2). Pairwise comparisons revealed significant differences only between the “after1” period and the “after2” and “after3” periods (Figure 2b). In comparison, P_1year_ was fairly similar among periods: it was 0.51 (CI: 0.39–0.68) before protection, increased to 0.57 (0.47–0.69) in the “after1” period, and then decreased again to 0.33 (0.25–0.43) in both the “after 2” and “after3” periods. In agreement, *M*_s_ was 369.4 days (CI: 240.3–NA) in the “before” period, increased to 467.3 days (295.5–NA) in the “after1” period, and then decreased to 219.0 days (175.4–282.3) in the “after2” period and 243.5 days (191.2–285.4) in the “after3” period.

### 3.2 Selection on behavioral traits

**FIGURE 3:**
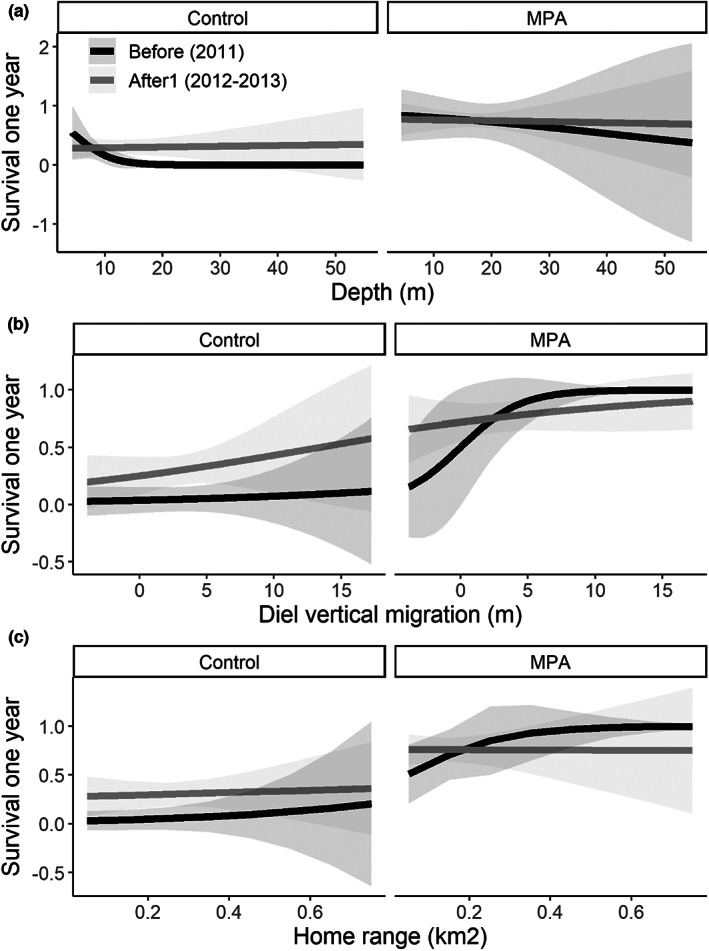
Relationship between survival and the different behavioral traits in the control area and the marine protected area (MPA) before and after MPA implementation. Post hoc test revealed that all slopes were nonsignificantly different from zero (all *p*‐values > 0.41). Pairwise comparisons of slopes within each site revealed no changes in slope from preprotection to protection (all *p*‐values > 0.15). Predictions were made for average levels of the other variables.

**FIGURE 4:**
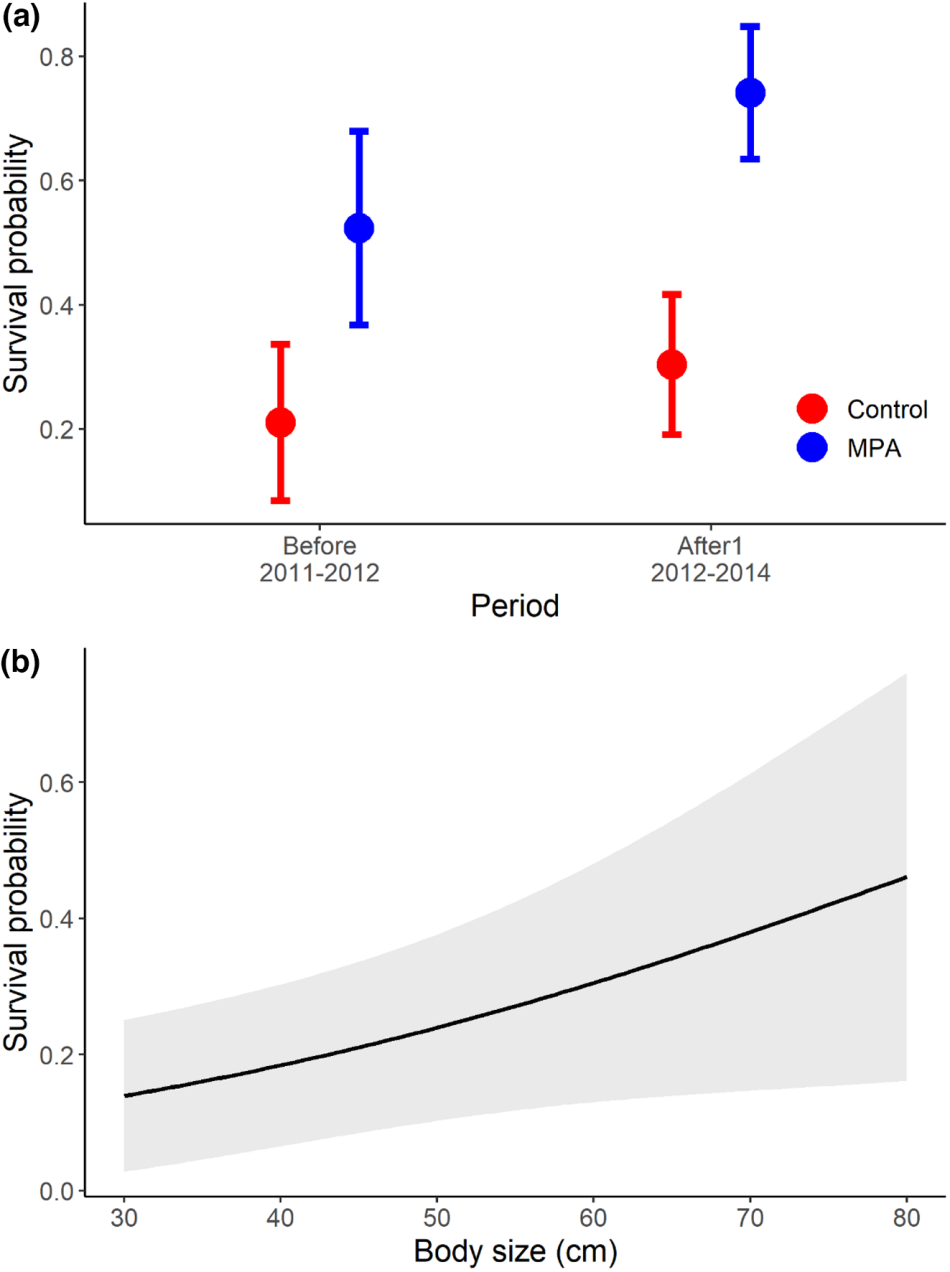
Results from the best binomial model investigating the effects of the period, body size, and behavior on the probability of surviving 1 year in the control and marine protected area (MPA). The only significant explanatory variables were period (suggesting an increase in survival in the marine protected area after protection) and body size. Predicted survival probabilities for the different periods (a) were calculated using a body size of 45 cm. Prediction for the range of body sizes (b) was calculated using the control area and the before period as reference levels.

The model selected for inference about drivers of survival did not include either the additive effect of any behavioral trait or any of the interactions between *Site_period* and behavior or body size (Table 2), suggesting no changes in selection acting on behavioral traits or body size after protection. Post hoc tests suggested that none of the slopes between survival and behavior was significantly different from zero, and no differences in slopes between the preprotection and the protection periods in any of the sites (Figure 3). The most parsimonious model did include an additive effect of *Site_period* and body size (Table 2). In particular, the probability of surviving the first year was higher in the MPA than in the control area. In addition, in the MPA, there was a significant increase in survival probability of 41.5% from “before” to “after 1” (pairwise comparison: *χ* ^2^ = 5.038, *df* = 1, *p*‐value = 0.025; Figure 4a). No difference in survival between periods was observed in the control area (*χ* ^2^ = 1.10, *df* = 1, *p*‐value = 0.295). Survival probability was higher for larger individuals regardless of the area and period considered (Figure 4b).

**TABLE 2:** Summary of the best binomial model investigating the effect of body size and behavior on the probability of surviving 1 year, considering the two periods for which both data from the control and the marine protected area (MPA) were available.

Term	Estimate	Std. error	*Z* value	*p*‐Value
Intercept	−1.27	0.383	−3.30	<0.001
Control_after1	0.494	0.471	1.05	0.294
MPA_before	1.42	0.499	2.84	0.005
MPA_after1	2.37	0.479	4.96	<0.001
Body size	0.322	0.147	2.18	0.029

*Note*: Before: cod tagged in 2011. After 1: cod tagged in 2012–2013.

**FIGURE 5:**
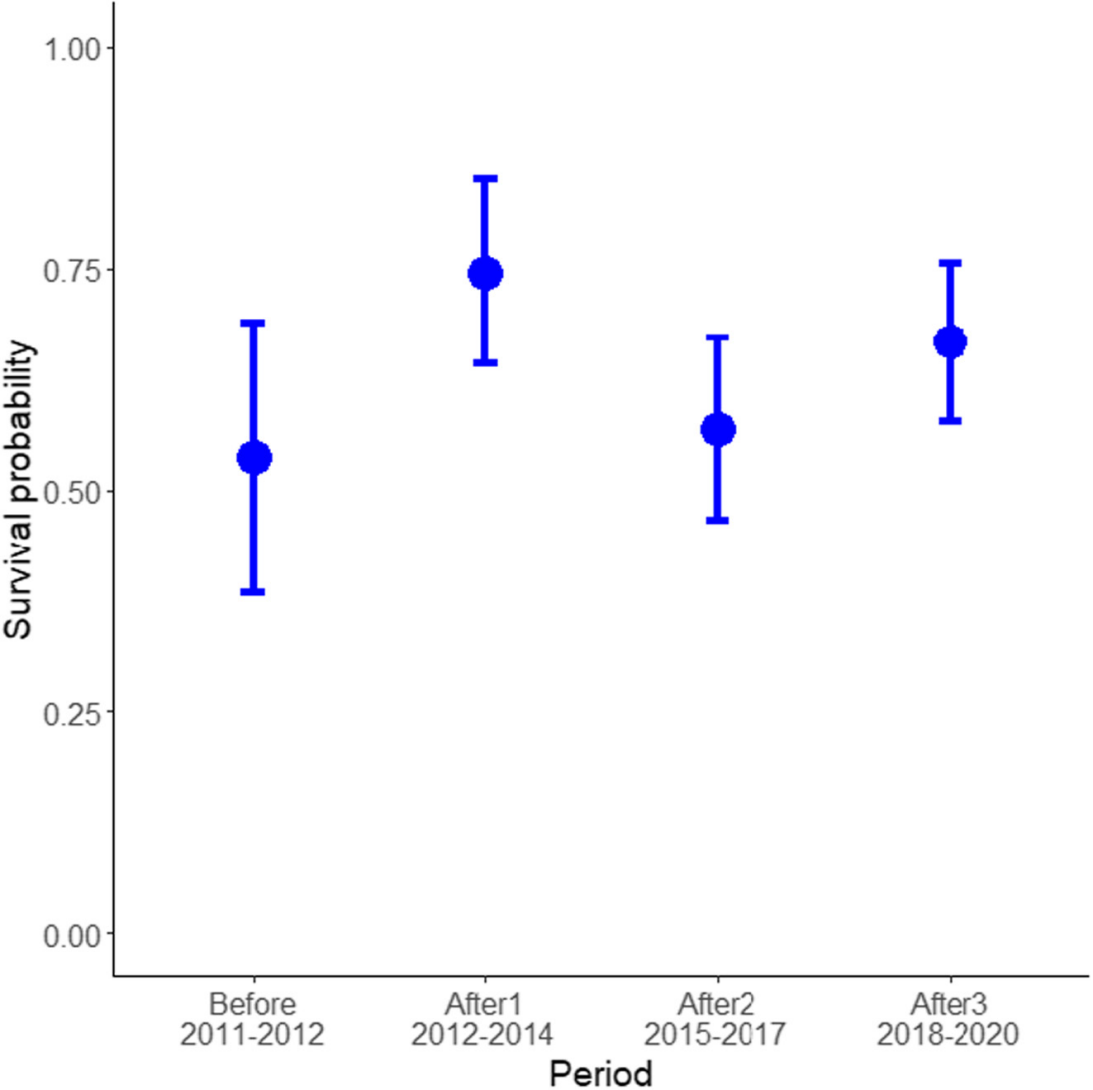
Effect of period on the probability of surviving 1 year in the marine protected area, as predicted from binomial generalized models.

When taking into account all the four periods for the MPA, we found that survival increased by 39.1% in period “after1” as compared to “before” (*p* = 0.027) but that increase was not maintained in periods “after2” and “after3” (Figure 5, Table 3).

**TABLE 3:** Summary of the optimal model investigating the effect of body size and behavior on the probability of surviving 1 year in the marine protected area, considering the four periods available

Term	Estimate	Std. error	*Z* value	*p*‐Value
Intercept	0.147	0.313	0.468	0.640
MPA_after1	0.932	0.421	2.22	0.027
MPA_after2	0.128	0.380	0.336	0.737
MPA_after3	0.547	0.374	1.46	0.144

### 3.3 Behavioral changes over time

**FIGURE 6:**
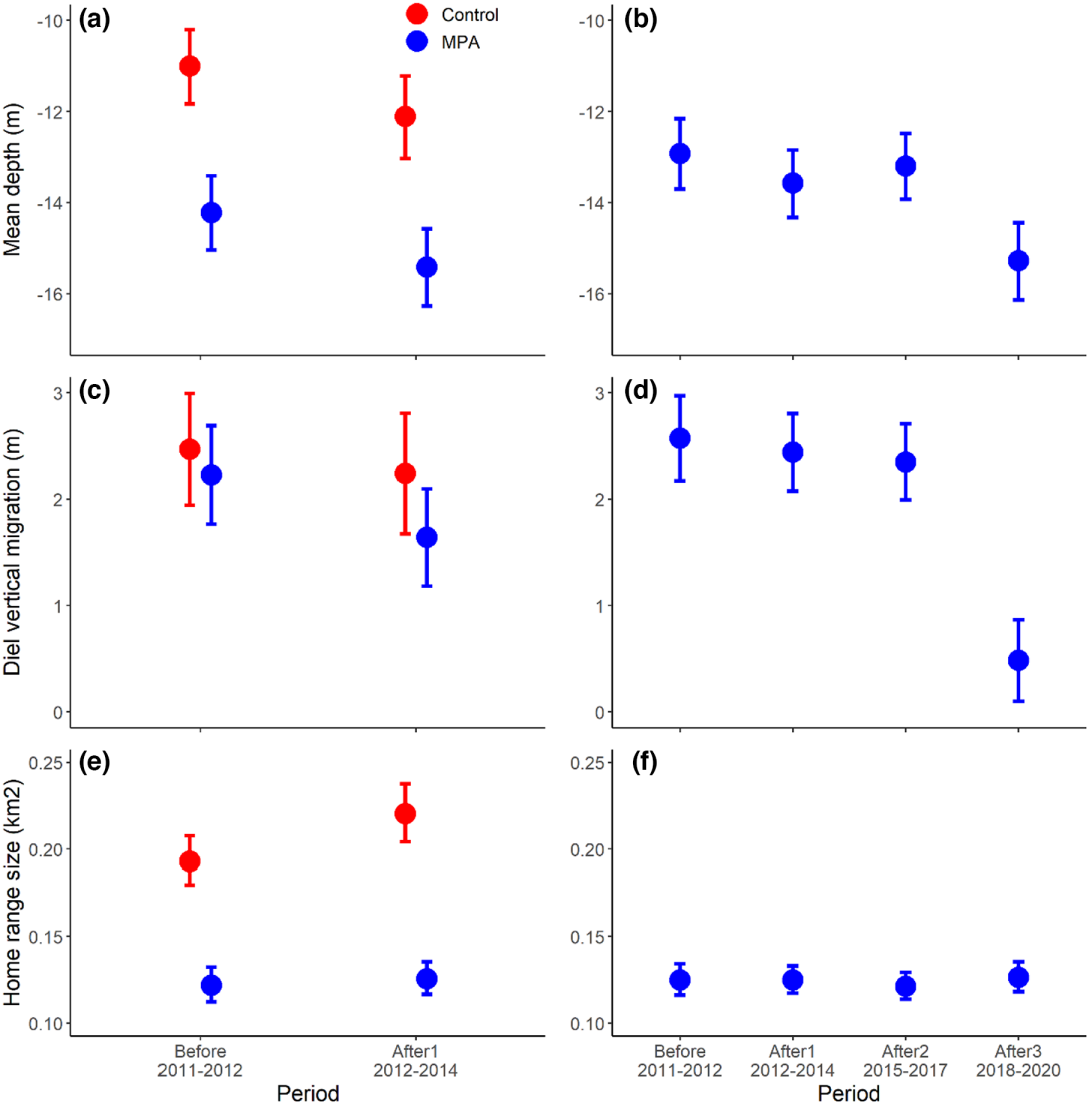
Variation of cod behavioral traits over the different periods before and after the implementation of the marine protected area (MPA), as predicted from mixed‐effect additive models. Plots on the left (a, c, e) correspond to the predictions from BACI analyses, i.e., from models considering both the control area and the MPA and just the “before” and “after1” periods; plots on the right (b, d, f) correspond to models only for the MPA that include two extra periods (“after2” and “after3”). On each panel, predictions were made for fixed values of other variables in the models (body size = 40 cm, day of the year = 120, week = 26).

Depth position varied over the year with maximum depths in summer and shallower depths in winter in both sites (Figure S4a). No effect of body size on depth position was found (Figure S4b, Table S1). On average, fish in the MPA stayed in deeper waters than fish in the control area (Table S1). In both the control area and the MPA, depth significantly increased from “before” to “after1” periods (Table S1, Figure 6a), but the rate of change was not significantly different between sites (interaction term period × array *p* = 0.888). When taking into account the four periods in the MPA, we observed a trend toward occupying deeper waters over time, with the deepest values observed in the period after3 (Figure 6b, Table S2).

The magnitude of the diel vertical migration varied over the year, with larger migrations displayed in mid‐September, and smaller migrations in mid‐April in both sites (Figure S4c). Also, smaller fish displayed larger migrations both in the control area and the MPA (Figure S4d). In general, the magnitude of the diel vertical migration was similar in the control area and the MPA (*p* = 0.211, Table S1), and it did not change significantly from “before” to “after1” (*p* = 0.108; Table S1, Figure 6c). When taking into account the four periods in the MPA we observed that the diel vertical migration decreased significantly in period “after3” compared with the other periods (Figure 6d, Table S2).

Home range size varied over the year in both sites although in a different way. In the control area, home range size was larger in winter and smaller in summer, while the opposite pattern was observed in the MPA (Figure S4e). Body size also had a contrasting effect between areas, with smaller fish having a larger home range in the control area but smaller in the MPA (Figure S4f). In general, home ranges were larger in the control area than in the MPA (*p* = 0.001, Table S2). In the control area, but not in the MPA, home range size increased from “before” to “after1” periods (interaction term period × array *p* = 0.021, Table S2, Figure 6e). When taking into account the four periods in the MPA we observed no changes in home range size over the four periods (Figure 6f, Table S3).

## 4 DISCUSSION

In this study, we monitored the survival and behavior of two cod populations in southern Norway. In one fjord, where one of these cod populations is found, a 1.5 km^2^ no‐take marine reserve and adjacent angling‐only buffer zones have been in place since 1 year after our study started. Although we observed a rapid increase in the annual survival of cod in this fjord right after MPA establishment, survival subsequently decreased to preprotection levels just 3 years after. Changes in selection patterns acting on behavioral phenotypes or body size were not observed. After almost a decade, the population within the MPA tended to occupy deeper waters and display smaller diel vertical migrations, although such changes cannot be unequivocally attributed to protection. Our results challenge previous expectations of fitness benefits of MPAs and reject the hypotheses of short‐term protection effects on fish behavior.

Cod survival was low in all of the observed scenarios. In scenarios with no protection, the probability of surviving 1 year ranged between 0.24 and 0.51, suggesting that a major part of the population (within the observed size range) suffered from mortality during just 1 year. Such strikingly high mortality rates of coastal cod were also reported in the past. Olsen et al. (2012) found that just 3 months after tagging, 45% of the individuals in the Sømskilen population had died, and Olsen and Moland (2011) reported harvest mortality of 42% after 6 months in a nearby population. Unexpectedly, we found no evidence for a lasting positive effect of protection on survival. While survival increased by ~40% right after MPA establishment, it decreased again after just 3 years reaching preprotection levels. In comparison, within the same study region, European lobster experienced a sharp increase in survival up to 3 years after protection followed by a marginal decrease after 9 years, where long‐term survival still remained higher than preprotection levels (Fernández‐Chacón et al., 2020). So why did cod not experience a similar survival benefit in the MPA? The fact that the marine reserve was rather small and surrounded by a buffer zone where angling is allowed implies that cod was still not fully protected from fishing (Villegas‐Ríos et al., 2021). In fact, in a previous study, we showed that although almost all cod individuals were tagged inside the FPA, most of them (~70%) spent some time outside the FPA boundaries (i.e., in the buffer or open areas) suggesting that this area is too small to effectively grant full‐time protection to this population (Villegas‐Ríos et al., 2021). Unfortunately, direct information on fishing pressure outside the MPA was not available, but it is plausible that anglers reacted to the implementation of the MPA with a rapid decrease in fishing pressure (i.e., increasing cod survival), and potentially returning to the buffer areas in later years (Alós & Arlinghaus, 2013). Also, some poaching does occur inside the FPA as suggested in a previous study (Villegas‐Ríos et al., 2021). Another possibility is that density‐dependent factors or ecosystem‐level changes, not accounted for in this study, regulate the survival of cod in the MPA. In fact, natural mortality can increase in scenarios of protection, mitigating the expected benefits of reduced fishing mortality (Swain, 2011). Alternatively, coastal cod in southern Norway may have been selected for rapid life histories following decades of intense exploitation (Espeland et al., 2008; Lund et al., 2011). Larger and longer‐lived genotypes may have been largely removed from the coastal areas, leaving behind phenotypes characterized by short lives, as expected from life history theory and previous empirical studies (Reznick et al., 1990). While cod life histories can be highly variable, cod in the coastal Skagerrak usually mature at an early age of 2 or 3 years (Kuparinen et al., 2016; Olsen et al., 2004). Under this scenario, the protection benefits granted by the MPA and the potential impact of behavioral traits on survival may simply not be assimilated, in the short term, by the populations. This possibility needs to be carefully assessed as recent research has suggested that phenotypic plasticity, and not genotypic change, is the reason for earlier maturity in Atlantic cod following intense fishing exploitation in the North Atlantic (Pinsky et al., 2021).

Our results suggest that the harvesting of cod was nonselective on behavioral traits. This contrasts with a previous study on the Sømskilen cod population, which found that harvesting with passive gears selected against individuals moving in shallow waters and displaying extensive diel vertical migration and consistent horizontal movements (Olsen et al., 2012). Given that the size of the tracked individuals was similar in both studies (30–65 cm in Olsen et al., 2012 and 30–72 cm in our study), the observed differences suggest that selection patterns acting on behavioral traits are not fixed over time. Selection might depend on other environmental factors not monitored in this study, but we also know that one important component of the fishery has changed in the time period between these two telemetry studies. Previously, there was a widespread commercial fishery for European eel using fyke nets in shallow water. This fishery also caught a substantial amount of cod as a bycatch (Fernández‐Chacón et al., 2017) but was closed in 2009 due to the depleted state of the eel population. Fyke nets are no longer allowed, neither for commercial or recreational fishers. Finally, the observed differences might be due to methodological aspects of the analyses. While Olsen et al. (2012) estimated survival over 3 months in summer, in our study survival was computed over the first year after tagging, allowing for more mortality events to happen and potentially diluting any selection effect on body size or behavioral traits.

Contrary to our expectations, we did not observe any selection pattern on behavioral traits initiated after MPA implementation. Previous studies suggested that protection may select against individuals with larger mobility as they have greater chances to expose themselves to the fishery outside (Baskett & Barnett, 2015; Parsons et al., 2010; Villegas‐Ríos, Moland, et al., 2017). In fact, in a previous study on the same MPA cod population, individuals with larger home ranges experienced higher fishing mortality outside the MPA (Villegas‐Ríos et al., 2021). Based on a more extensive dataset, the current study found no overall selection against more mobile phenotypes after protection. One reason for that could be, in principle, that fishing pressure outside Tvedestrand MPA is relatively low and its impact on the overall selection patterns relatively weak compared with natural mortality. However, this seems unlike since an extensive mark‐recapture study has shown that fishing mortality on the Skagerrak coastal cod is generally very high and—especially for larger individuals—much higher than natural mortality (Fernández Chacón et al., 2015). Alternatively, selection could be maintained by continued, or intensified, fishing in the buffer zones and also poaching inside the FPA. Such effects could not be investigated separately because fish moving in the buffer areas and the FPA often refer to the same individuals, as explained above.

We did not detect any significant change in the mean behavioral phenotypes of the MPA population over time that we could unequivocally assign to a protection effect. For the time period for which data from both the control area and the MPA were available, the observed changes in mean depth use and diel vertical migration followed the same pattern in both study sites, suggesting that changes are not related to protection. However, longer‐term changes in the MPA toward the use of deeper waters and the display of smaller diel vertical migrations were observed, especially in the final period (2018–2020). Unfortunately, the lack of data from the control area in the mid‐term hinders us to conclude the causes of the observed trend. The short‐term changes in home range size observed in the control area were not mirrored in the MPA. It should also be noticed that the time series of “before” data available is relatively short (1.5 years) given the inter‐annual variation in movement traits, and a longer “before” time series would be better to establish the before‐protection baseline.

In conclusion, we have shown that fish survival and behavioral responses to MPA establishment may be less straightforward than previously anticipated. Protection effects on survival and behavioral traits relevant to conservation may depend on aspects typically not assessed in monitoring programs, such as density dependence, environmental parameters, MPA compliance, changes in fishing pressure, dispersal of early life stages or evolutionary constrains to life histories and behavior. Our results therefore highlight the need for appropriately scaled management experiments and more integrated approaches to understand the effects of intended protection on harvested aquatic species.

## 5 CONFLICT OF INTEREST

The authors declare no conflict of interests.

## Supporting information


Table S1
Table S2
Figure S1
Figure S2
Figure S3
Figure S4

## Data Availability

The data that support the findings of this study are openly available in Dryad Digital Repository at https://doi.org/10.5061/dryad.5x69p8d6n.
