# Supplementary material for: Eco‐evolutionary dynamics of Atlantic cod spatial behavior maintained after the implementation of a marine reserve
Source: Evol Appl. 2022 Oct 9;15(11):1846–58. doi: 10.1111/eva.13483 (PMC9679232; doi:10.1111/eva.13483)

**Supplementary material**

**Supplementary Table 1.** Results of the full generalized additive models investigating changes in behavioral traits over time using data from both the control site and the marine protected area (MPA). Only periods before and after1, corresponding to years 2011-2014, were considered in these analyses**.** doy=day of the year.

| DEPTH |  |  |  |  |
| --- | --- | --- | --- | --- |
| **Parametric** | **Estimate** | **Std. Error** | **t-value** | **p-value** |
| (Intercept) | -3.13 | 0.05 | -56.63 | <0.001 |
| period_after1 | -0.16 | 0.034 | -4.75 | <0.001 |
| site_MPA | -0.35 | 0.075 | -4.64 | <0.001 |
| period_after1:site_MPA | 0.006 | 0.047 | 0.14 | 0.888 |
| **Smooth terms** | **edf** | **Ref.df** | **F** | **p-value** |
| s(doy):site_ control | 1.946 | 2 | 78.307 | <0.001 |
| s(doy):site_MPA | 1.995 | 2 | 697.485 | <0.001 |
| s(Body size):site_ control | 1.001 | 1.001 | 0.023 | 0.880 |
| s(Body size):site_MPA | 1.001 | 1.001 | 0.613 | 0.434 |
| s(ID) | 277.337 | 350 | 5.757 | <0.001 |
|  |  |  |  |  |
| DIEL VERTICAL MIGRATION |  |  |  |  |
| **Parametric** | **Estimate** | **Std. Error** | **t-value** | **p-value** |
| (Intercept) | 2.267 | 0.250 | 9.050 | <0.001 |
| period_after1 | -0.228 | 0.168 | -1.360 | 0.174 |
| site_MPA | 0.431 | 0.344 | 1.251 | 0.211 |
| period_after1:site_MPA | -0.361 | 0.225 | -1.609 | 0.108 |
| **Smooth terms** | **edf** | **Ref.df** | **F** | **p-value** |
| s(doy):site_ control | 1.978 | 2 | 276.61 | <0.001 |
| s(doy):site_MPA | 1.995 | 2 | 964.93 | <0.001 |
| s(Body size):site_ control | 1.002 | 1.002 | 32.84 | <0.001 |
| s(Body size):site_MPA | 1.001 | 1.001 | 24.13 | <0.001 |
| s(ID) | 309.166 | 352 | 13.02 | <0.001 |
|  |  |  |  |  |
| HOME RANGE |  |  |  |  |
| **Parametric** | **Estimate** | **Std. Error** | **t-value** | **p-value** |
| (Intercept) | -1.753 | 0.036 | -48.013 | <0.001 |
| period_after1 | 0.132 | 0.032 | 4.112 | <0.001 |
| site_MPA | -0.189 | 0.058 | -3.222 | 0.001 |
| period_after1:site_MPA | -0.102 | 0.0439 | -2.314 | 0.021 |
| **Smooth terms** | **edf** | **Ref.df** | **F** | **p-value** |
| s(doy):site_control | 1.618 | 2 | 6.473 | 0.007 |
| s(doy):site_MPA | 1.985 | 2 | 189.896 | <0.001 |
| s(Body size):site_control | 1 | 1 | 5.359 | 0.020 |
| s(Body size):site_MPA | 2.798 | 2.827 | 3.396 | 0.009 |
| s(ID) | 255.15 | 354 | 3.785 | <0.001 |

**Supplementary Table 2.** Results of the full binomial models investigating changes in behavioral traits over time in the marine protected area (MPA) using the four periods available (before, after1, after2, after3). doy=day of the year.

| DEPTH |  |  |  |  |
| --- | --- | --- | --- | --- |
| **Parametric** | **Estimate** | **Std. Error** | **t-value** | **p-value** |
| Intercept | -3.37 | 0.051 | -66.040 | <0.001 |
| period_after1 | -0.090 | 0.033 | -2.720 | 0.007 |
| period_after2 | -0.038 | 0.047 | -0.797 | 0.425 |
| period_after3 | -0.314 | 0.059 | -5.294 | <0.001 |
| **Smooth terms** | **edf** | **Ref.df** | **F** | **p-value** |
| s(doy) | 1.996 | 2 | 1179.885 | <0.001 |
| s(Body size) | 1.001 | 1.001 | 0.486 | 0.486 |
| s(ID) | 342 | 397 | 10.526 | <0.001 |
|  |  |  |  |  |
| DIEL VERTICAL MIGRATION | |  |  |  |
| **Parametric** | **Estimate** | **Std. Error** | **t-value** | **p-value** |
| Intercept | 2.864 | 0.1953 | 14.669 | <0.001 |
| period_after1 | -0.1313 | 0.153 | -0.858 | 0.391 |
| period_after2 | -0.2219 | 0.199 | -1.110 | 0.267 |
| period_after3 | -2.0891 | 0.232 | -8.997 | <0.001 |
| **Smooth terms** | **edf** | **Ref.df** | **F** | **p-value** |
| s(doy) | 1.996 | 2 | 1181.36 | <0.001 |
| s(Body size) | 1.001 | 1.001 | 30.43 | <0.001 |
| s(ID) | 346.910 | 398 | 11.37 | <0.001 |
|  |  |  |  |  |
| HOME RANGE |  |  |  |  |
| **Parametric** | **Estimate** | **Std. Error** | **t-value** | **p-value** |
| Intercept | -1.965 | 0.0368 | -53.417 | <0.001 |
| period_after1 | 0.0004 | 0.028 | -0.017 | 0.987 |
| period_after2 | -0.030 | 0.036 | -0.825 | 0.409 |
| period_after3 | -0.013 | 0.041 | 0.310 | 0.775 |
| **Smooth terms** | **edf** | **Ref.df** | **F** | **p-value** |
| s(doy) | 1.987 | 2 | 257.270 | <0.001 |
| s(Body size) | 2.808 | 2.837 | 3.686 | 0.010 |
| s(ID) | 306.943 | 395 | 5.133 | <0.001 |

**Supplementary Figure 1.** Length-frequencies of tagged individuals by area (control and marine protected area [MPA]) and year tagged.


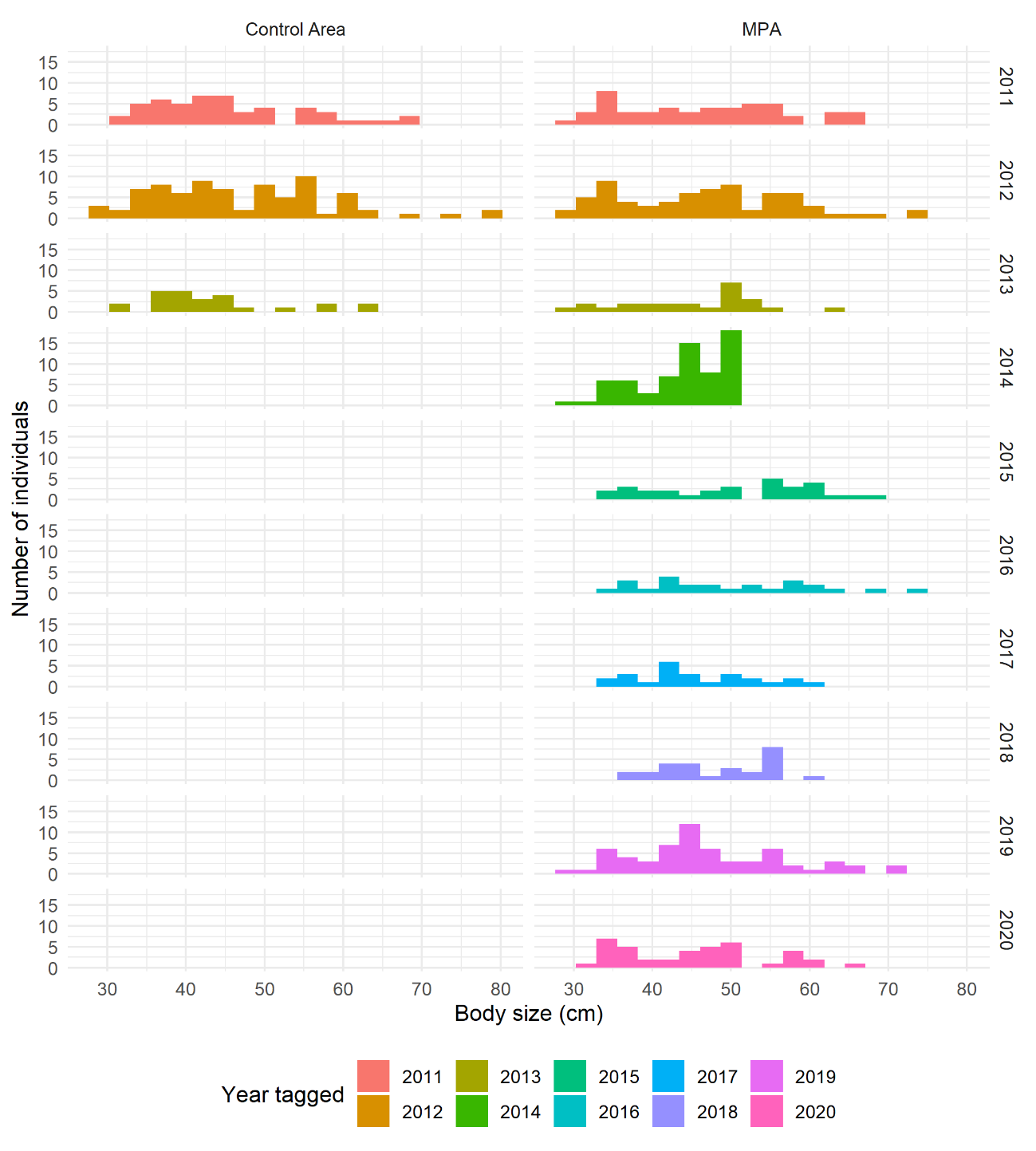


**Supplementary Figure 2.** Presence-absence plots of all the individuals tagged in this study. Plots are presented by area (control and marine protected area [MPA]) and by tagging year, to facilitate the visualization. Detection gaps happening at the same time for all fish correspond to dates of data download and receiver maintenance, when receivers stay out of the water for a couple of days.
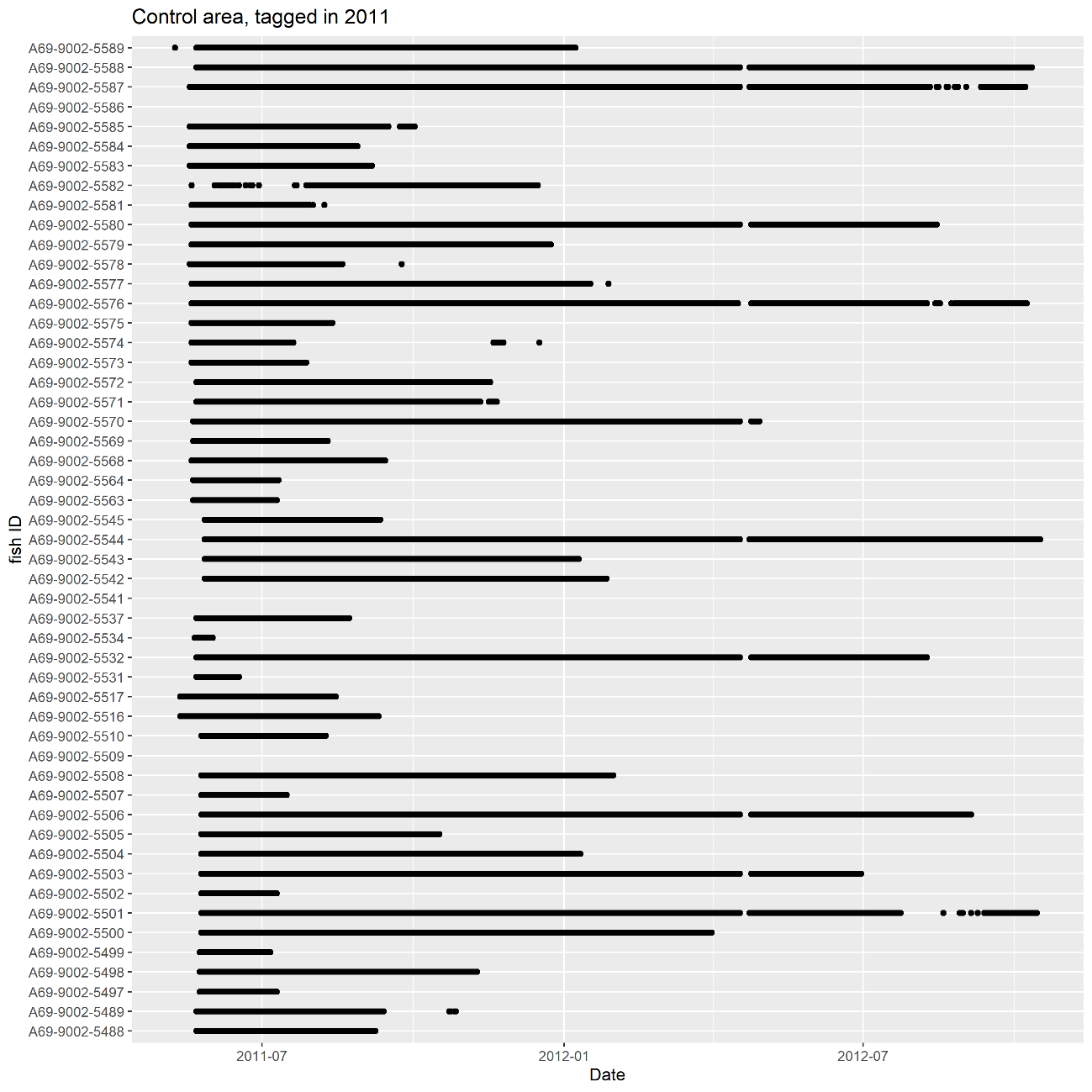

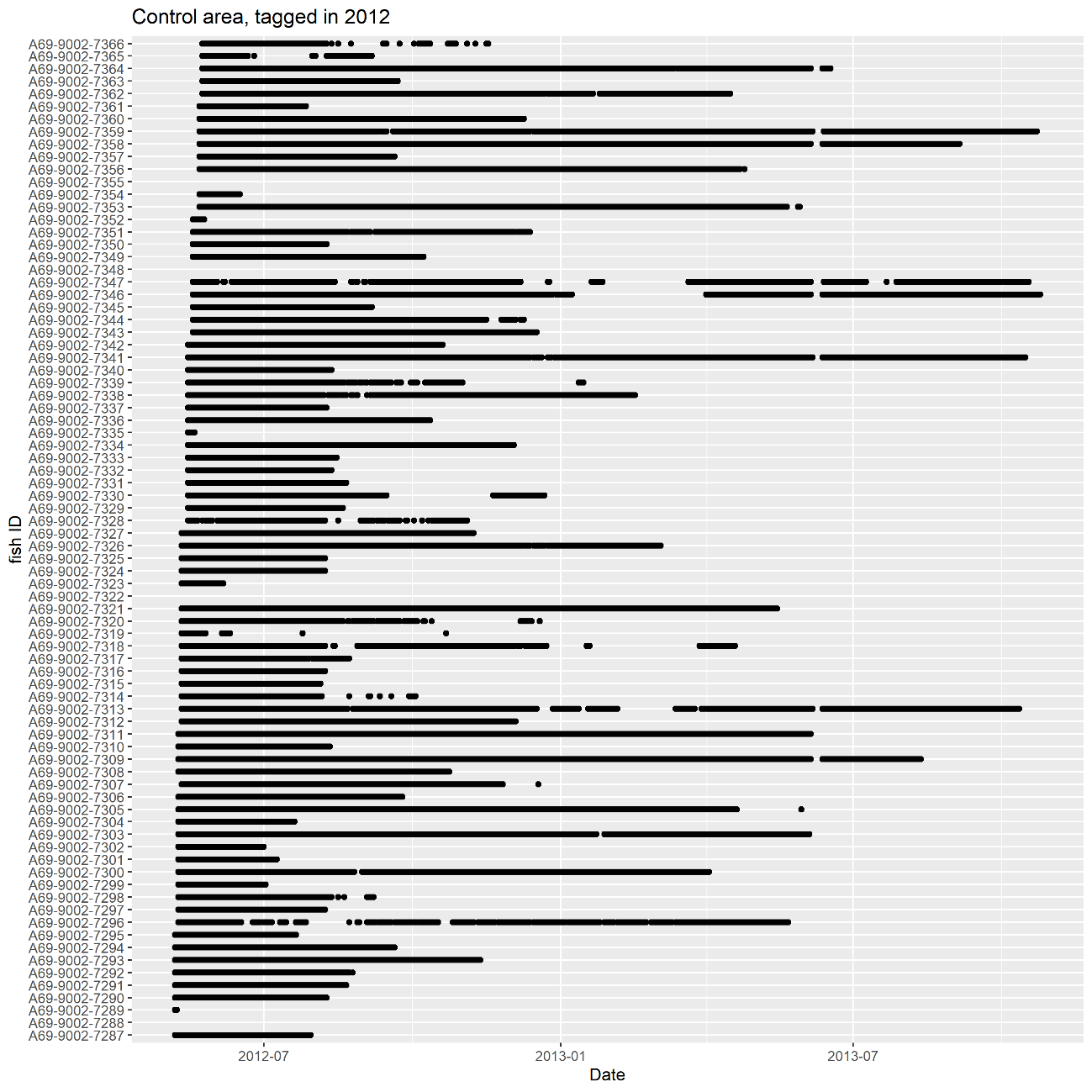

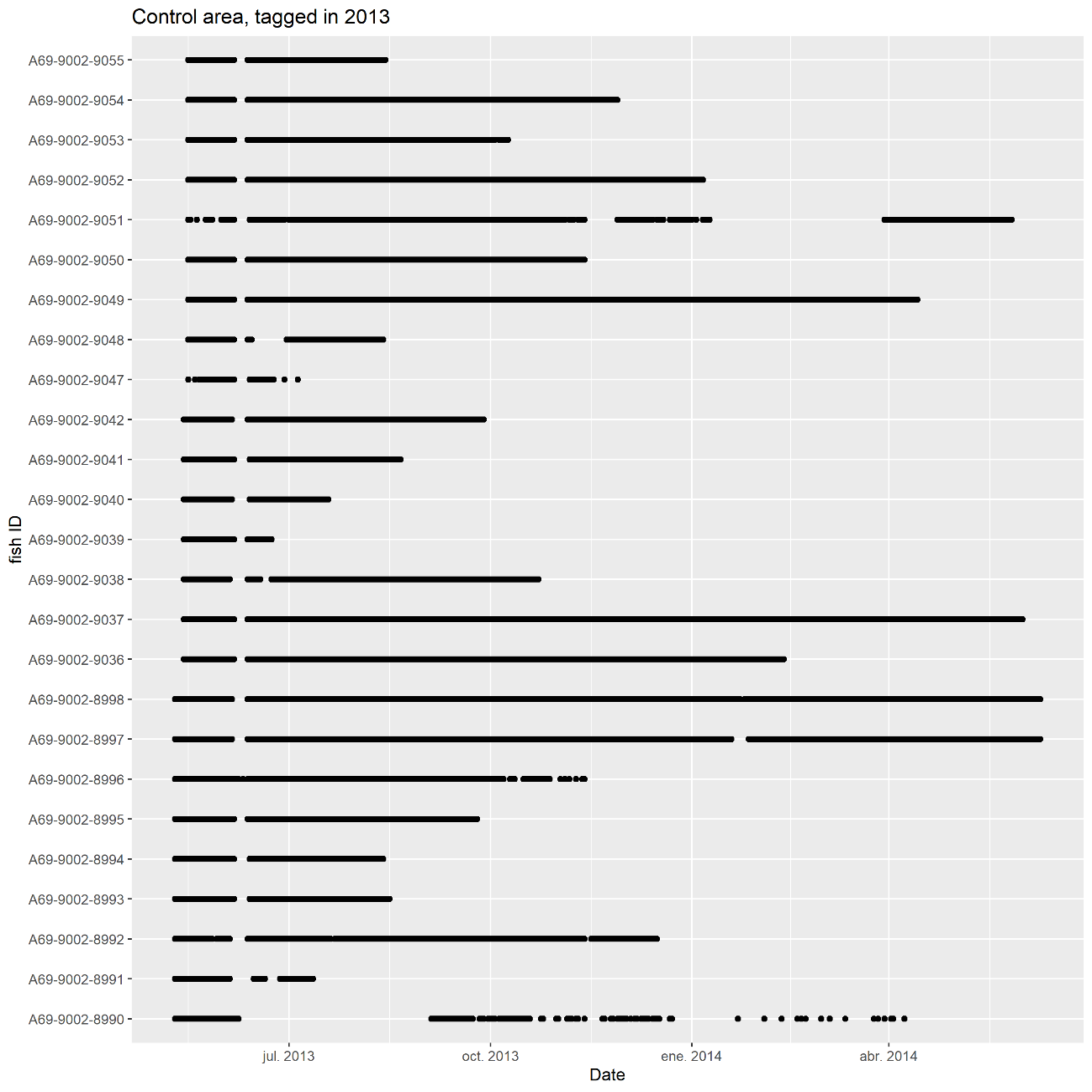

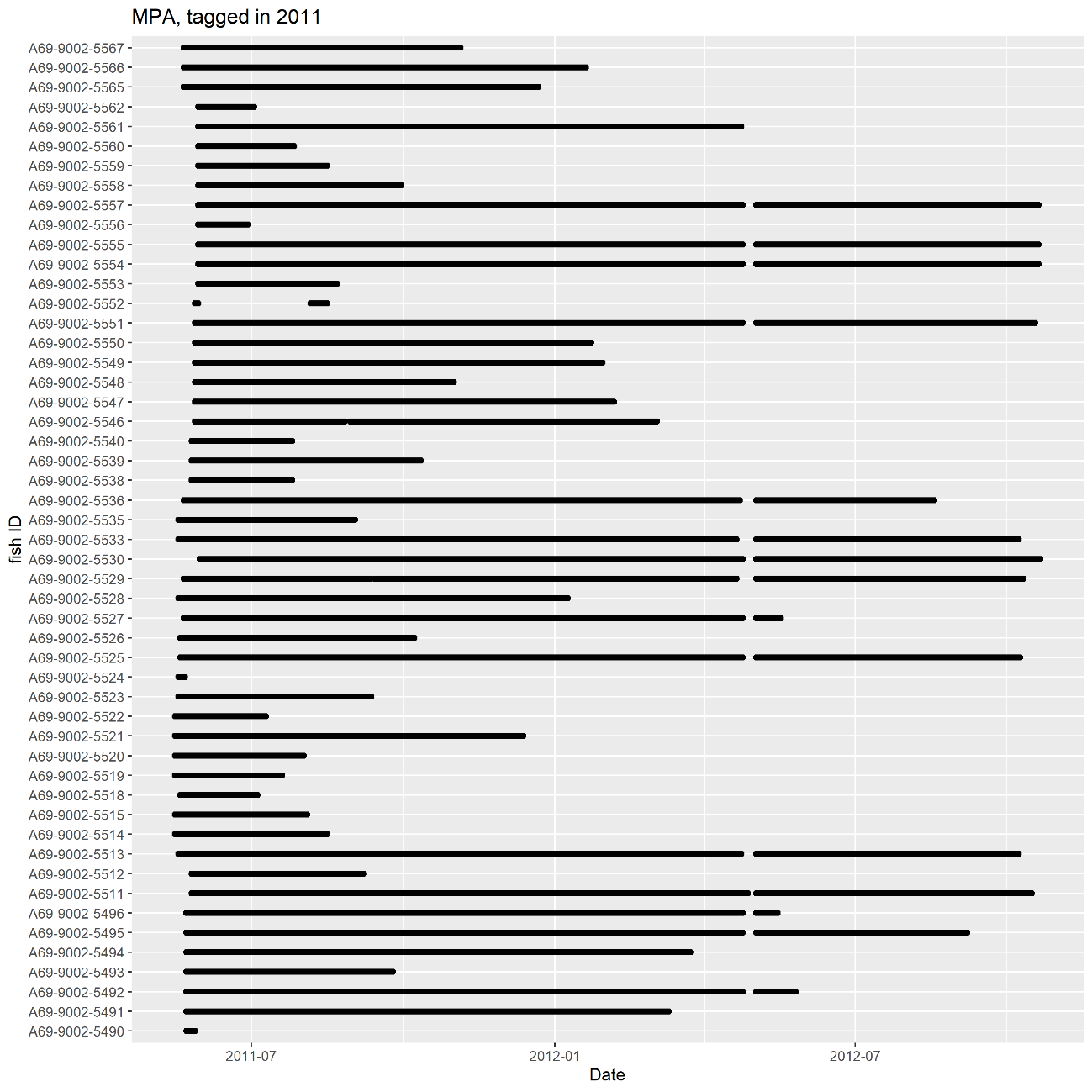

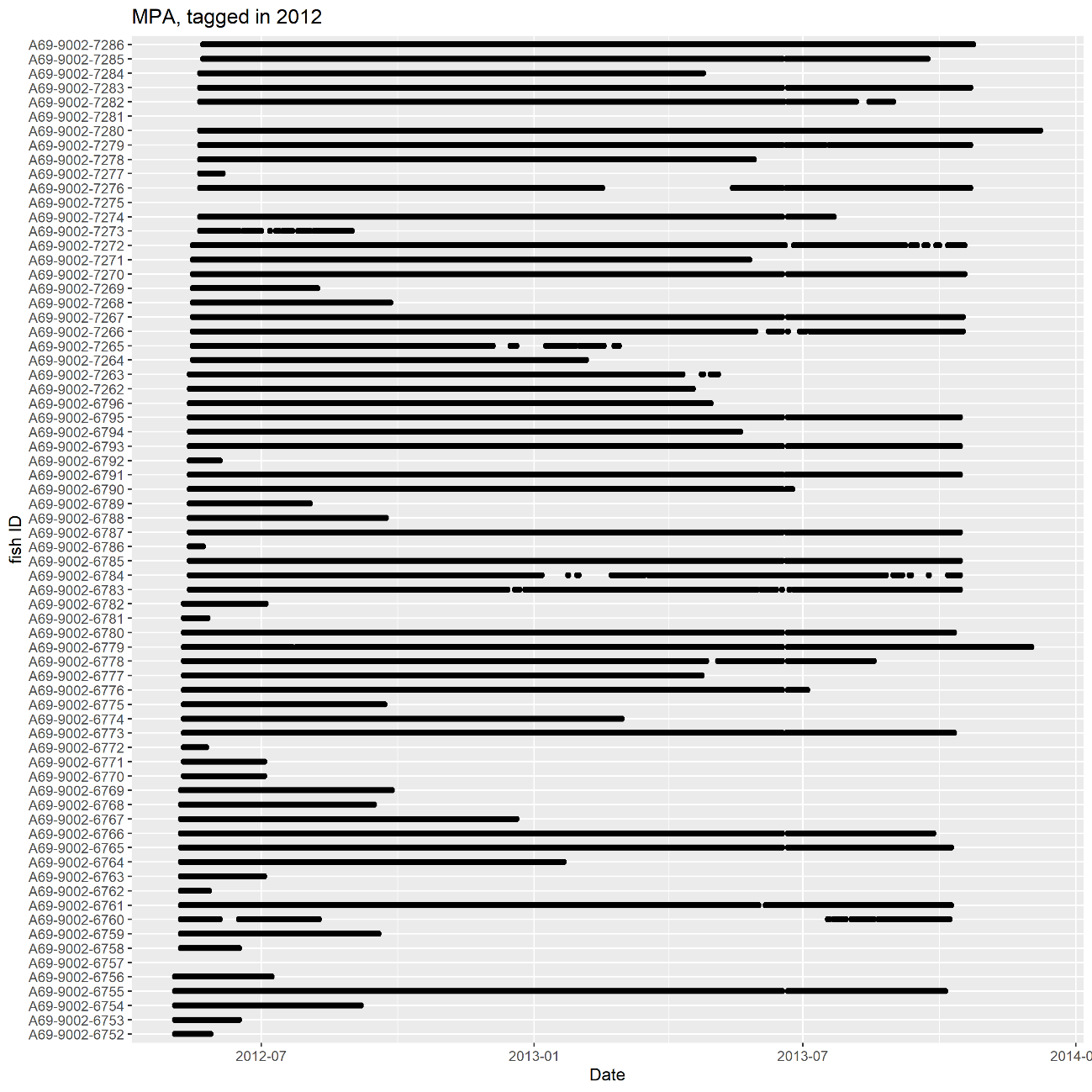

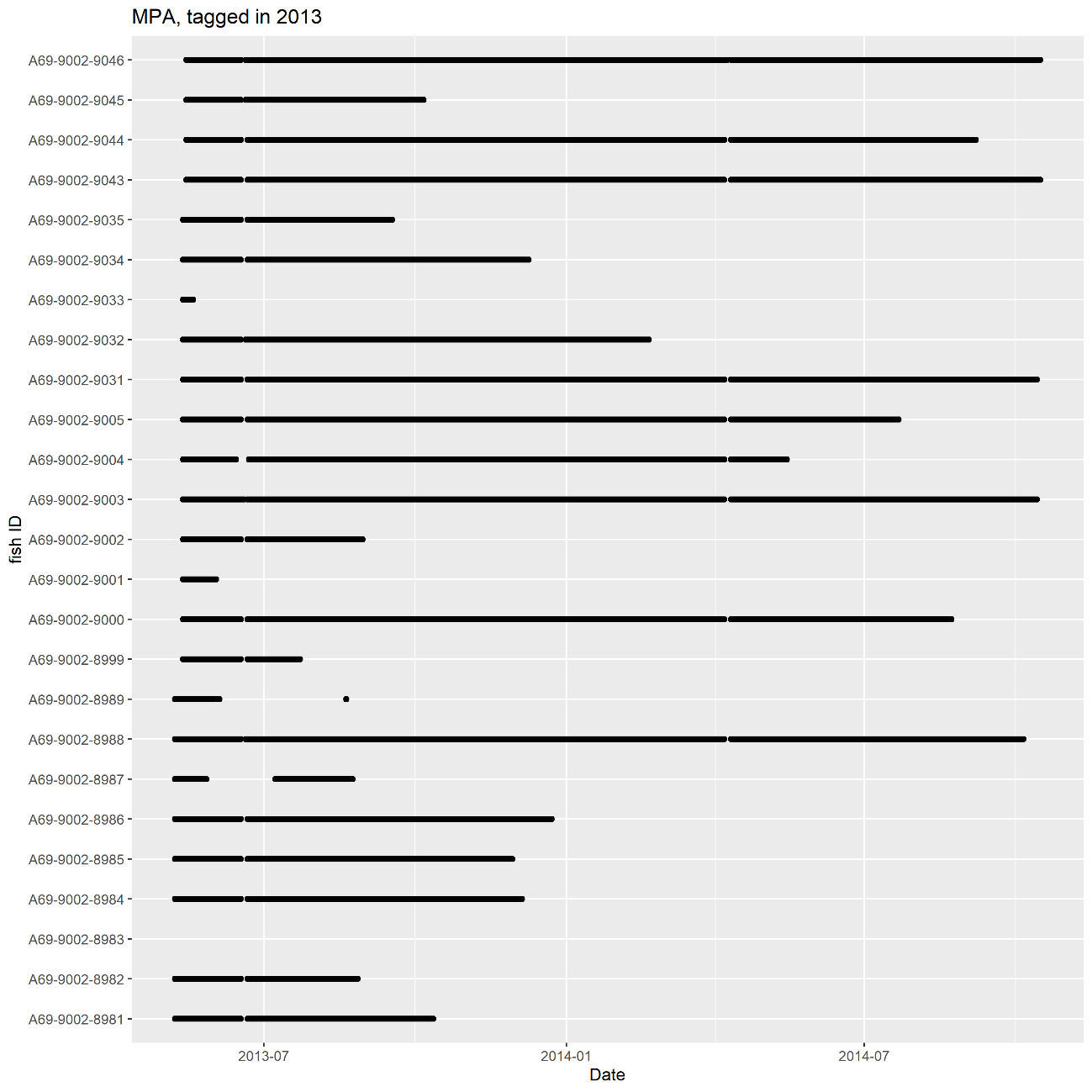

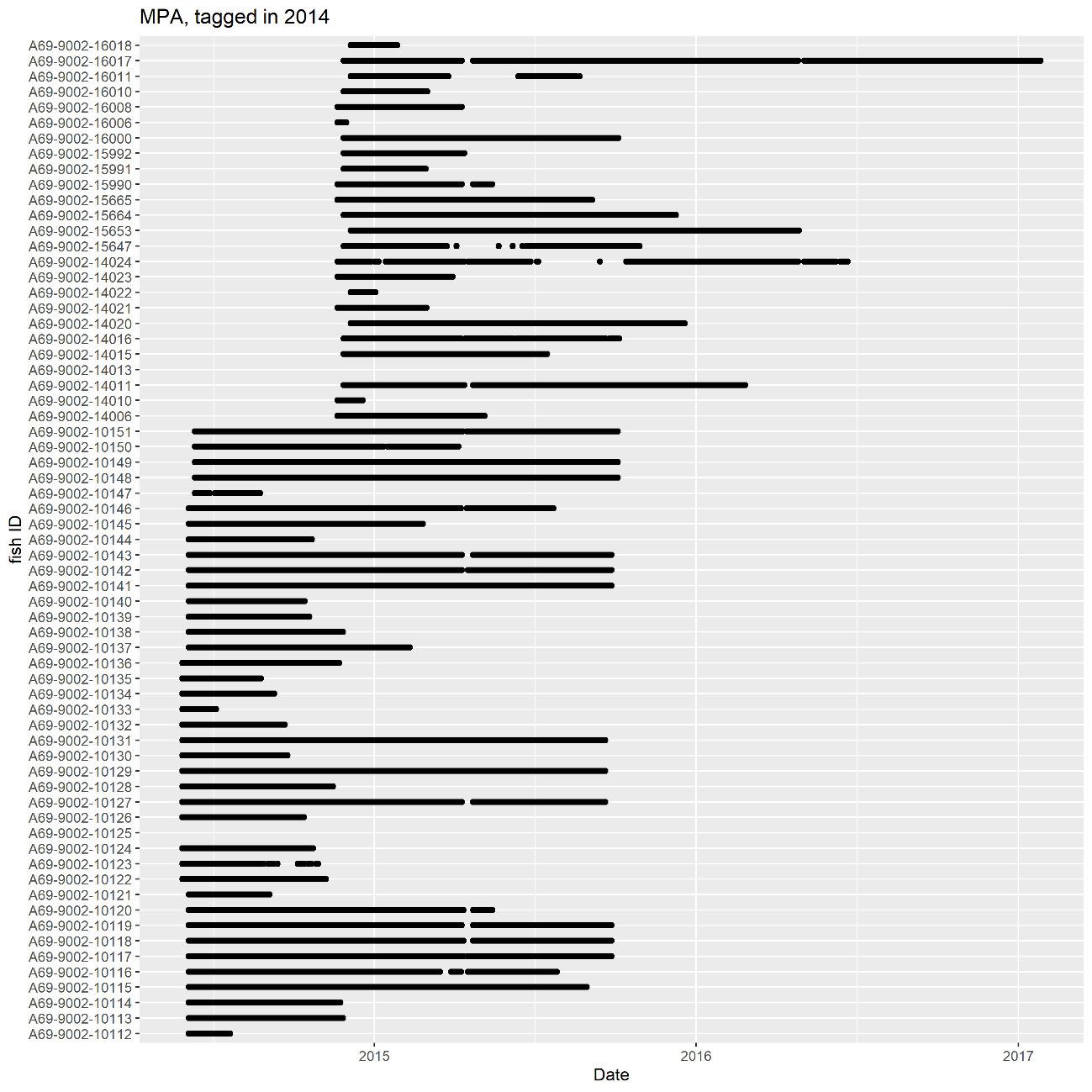

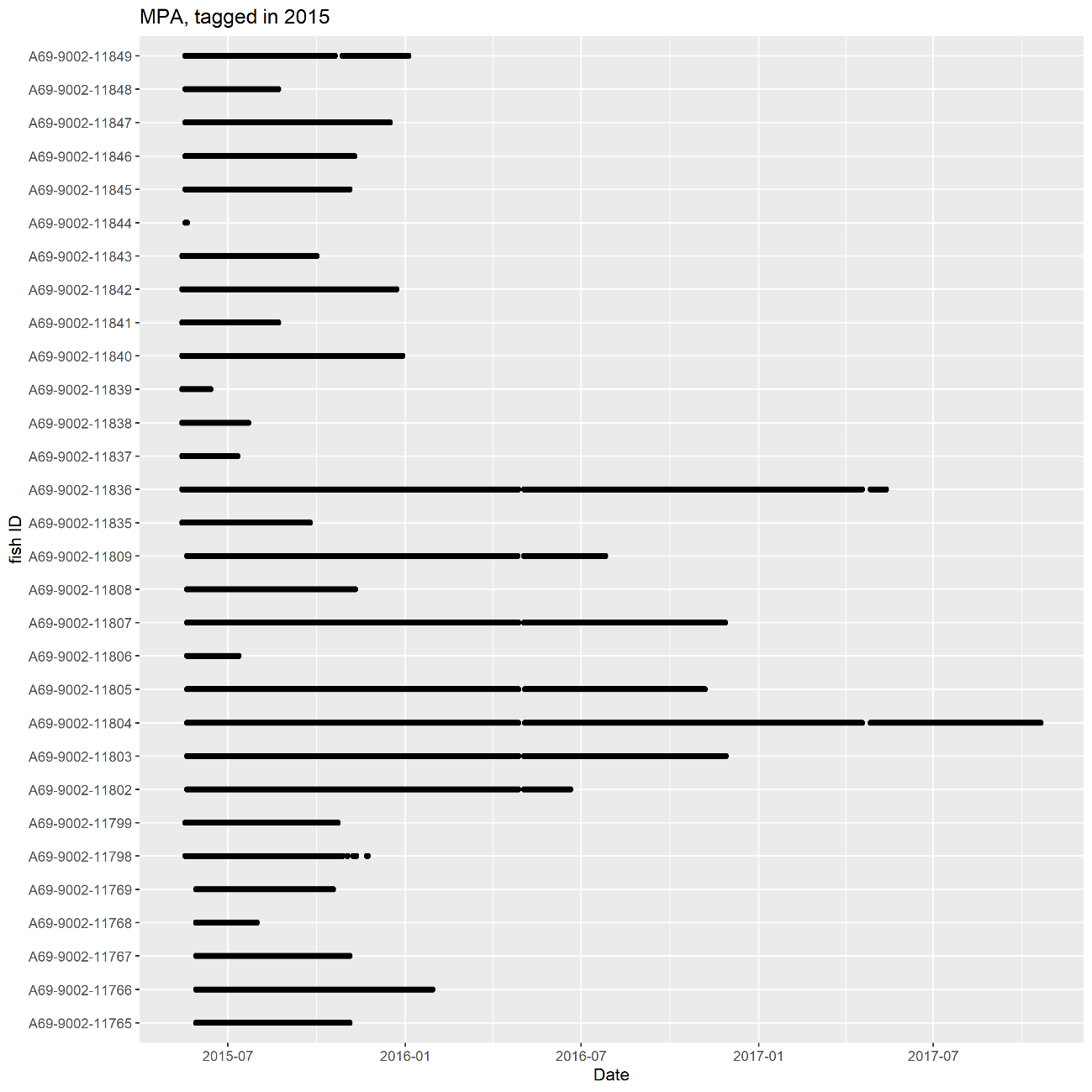

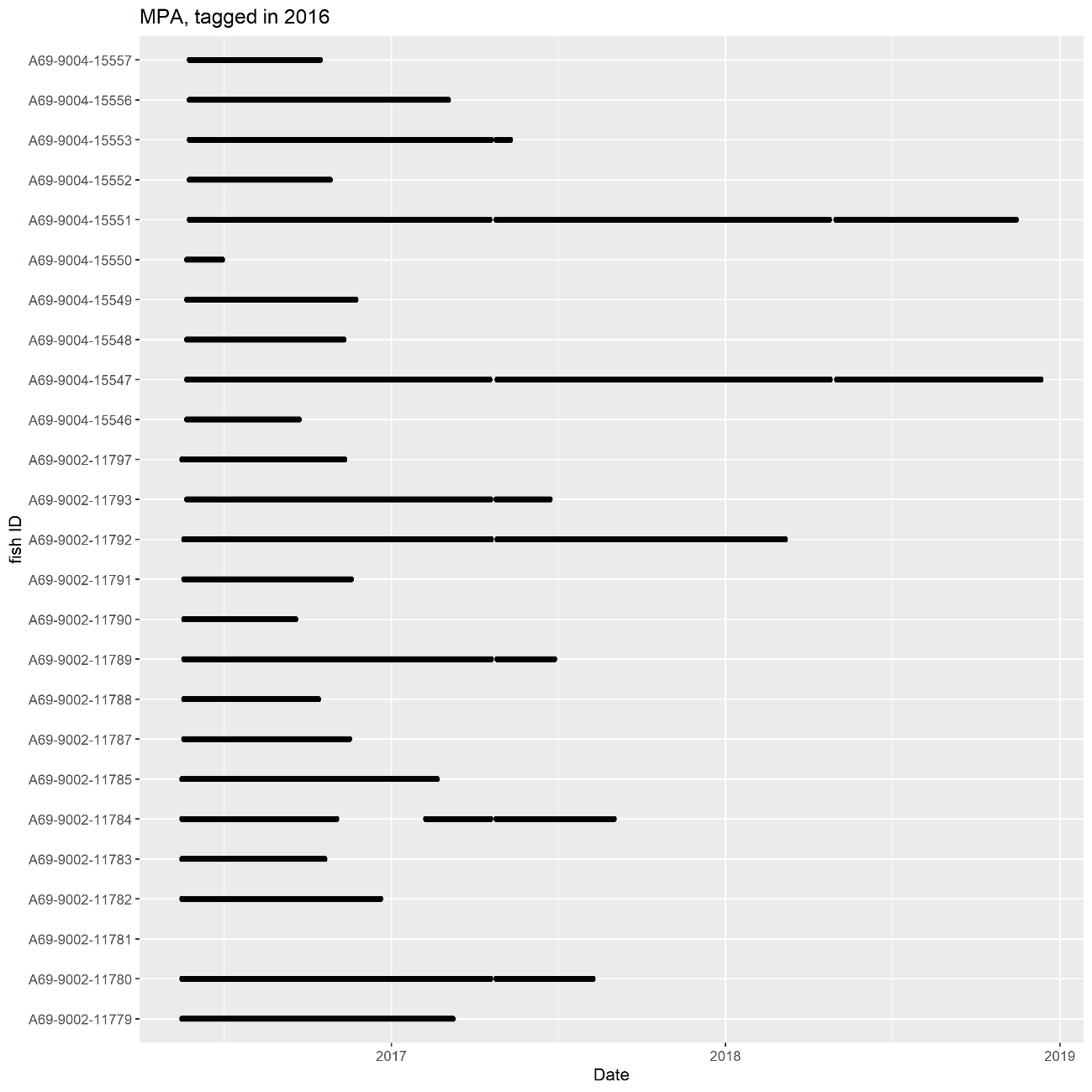

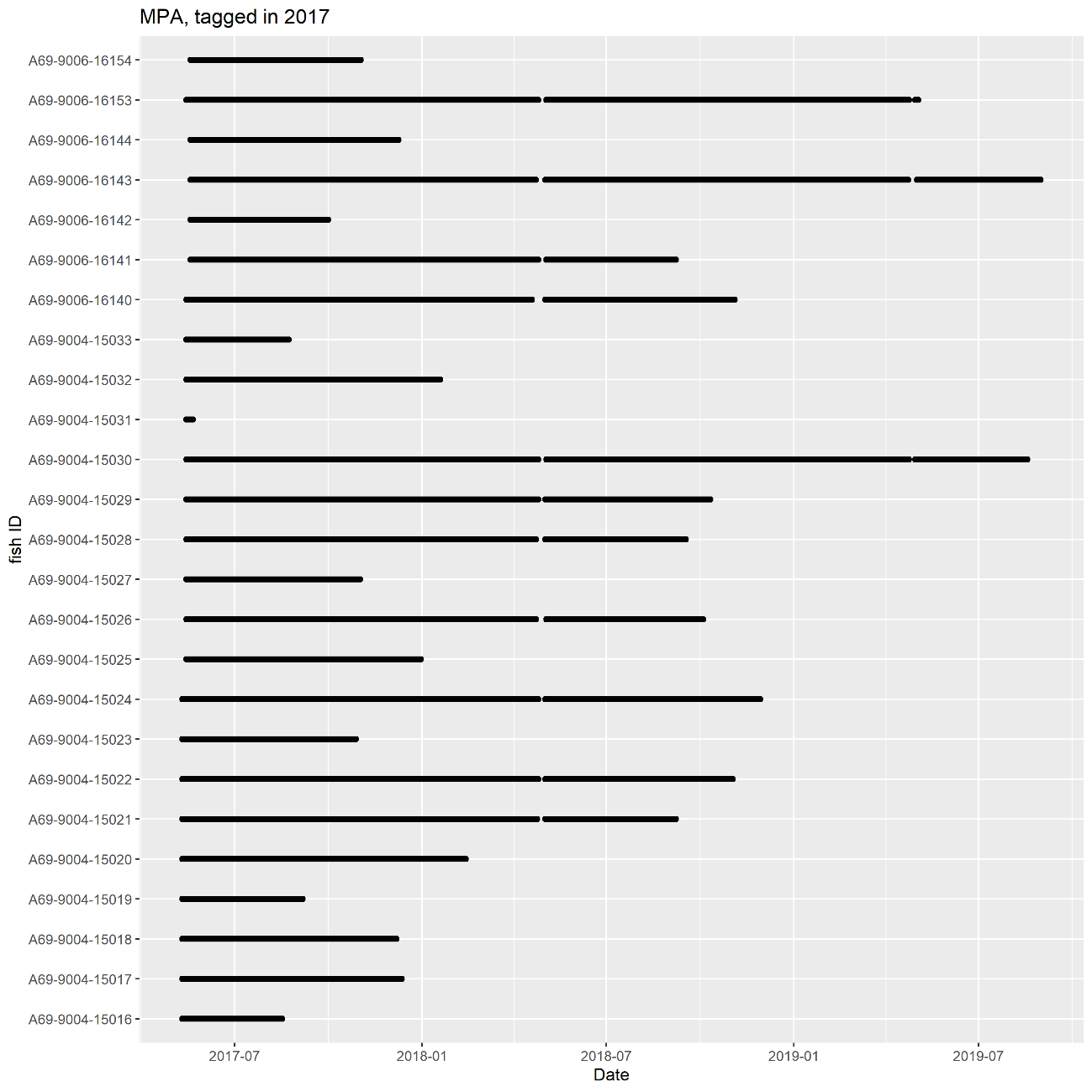

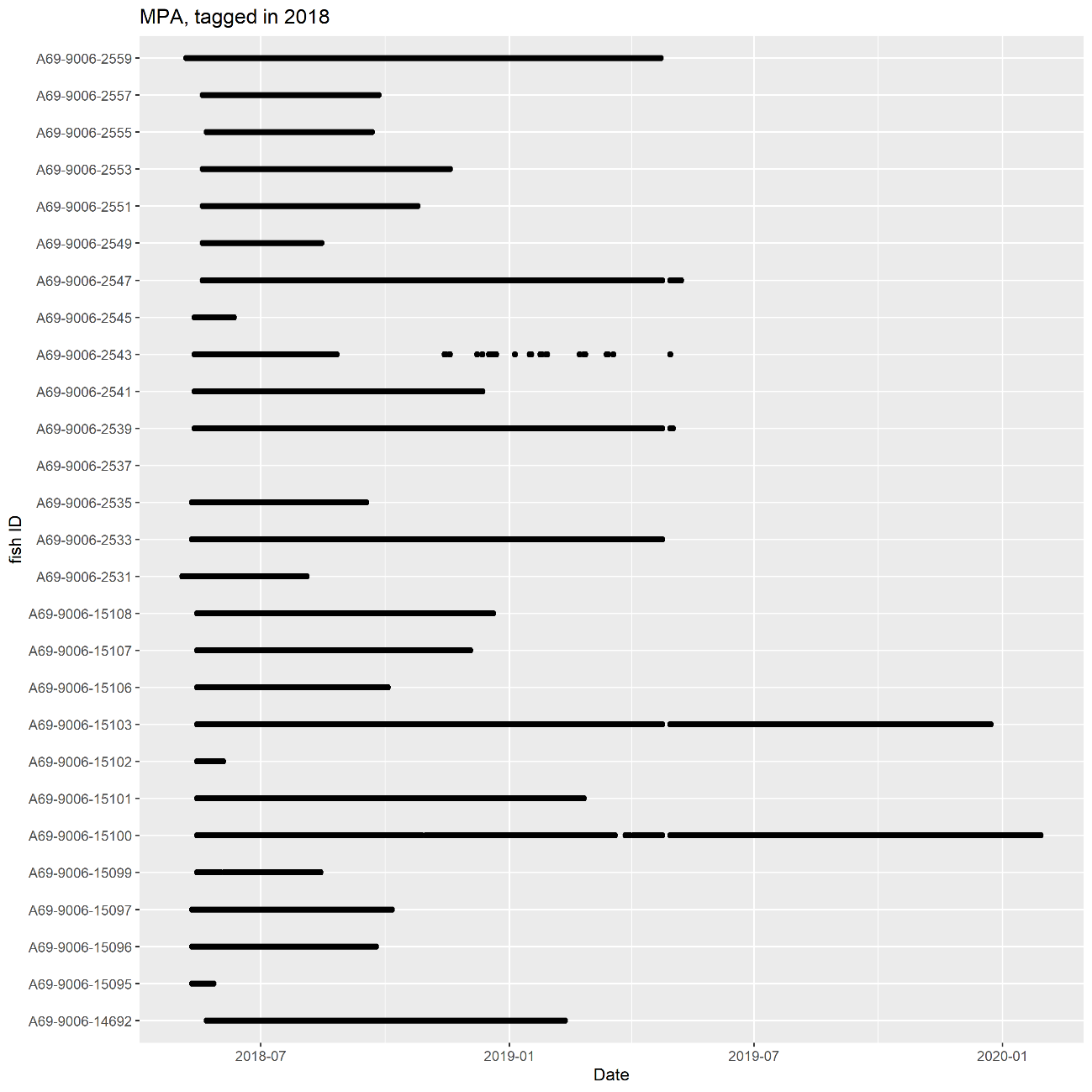

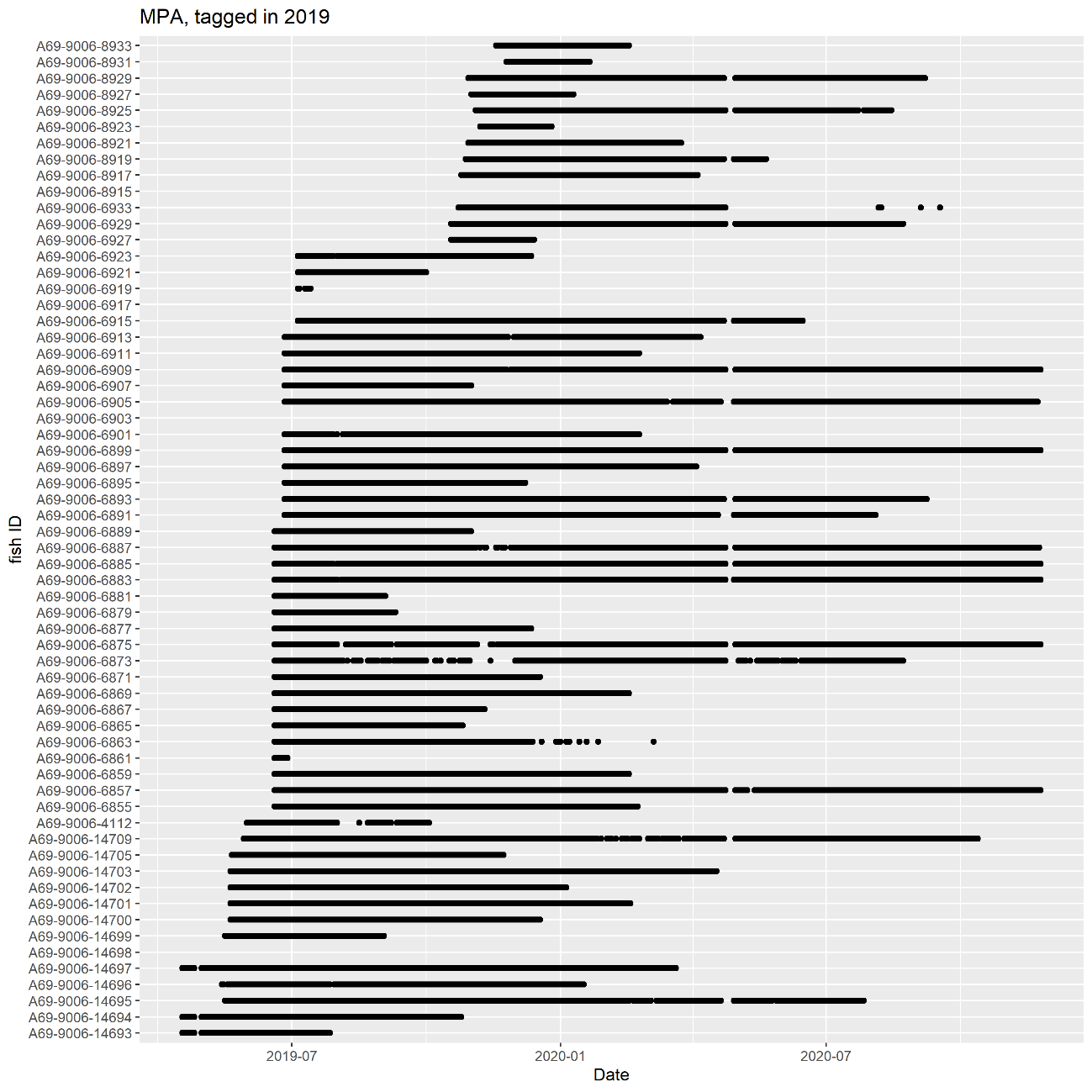

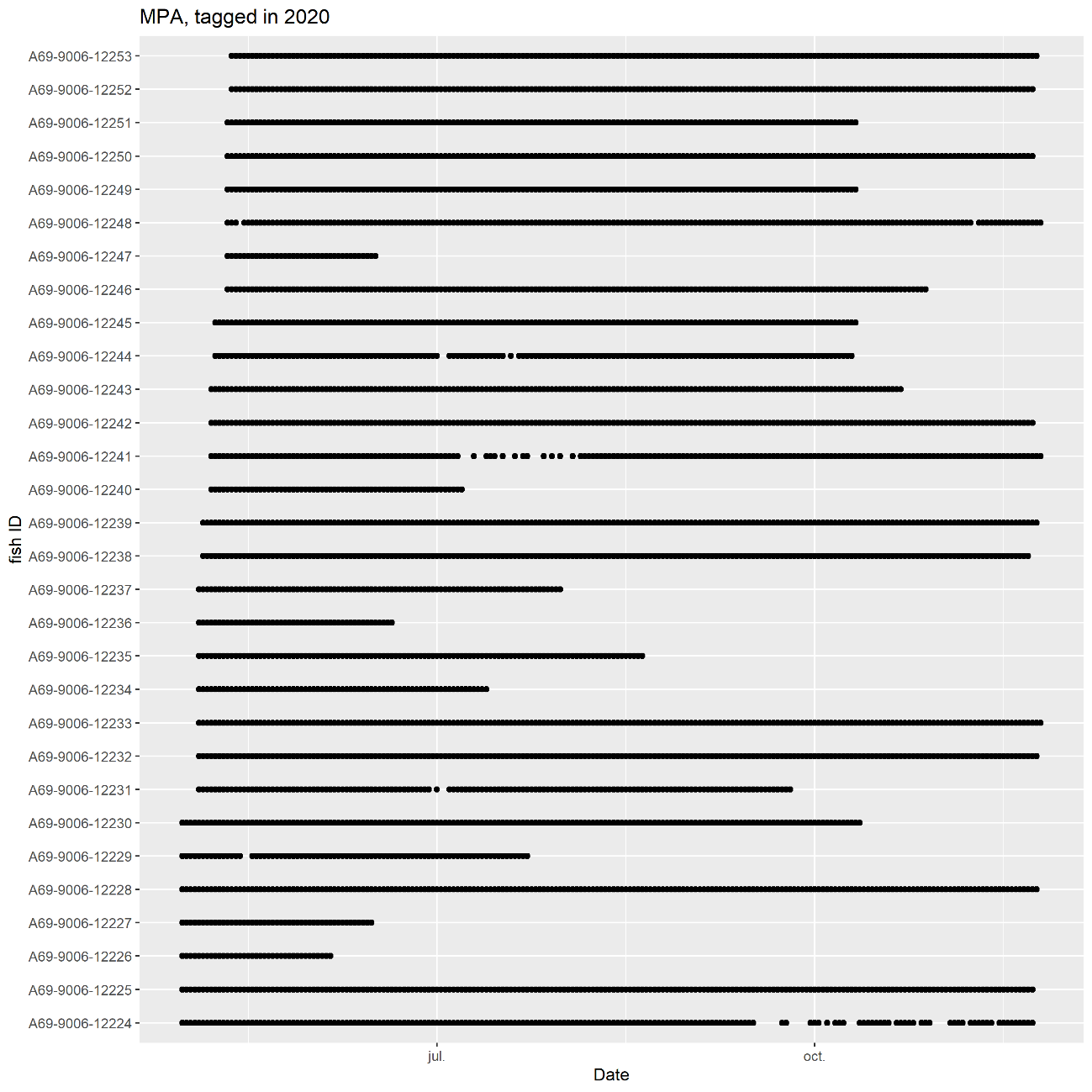


**Supplementary Figure 3.** Histograms showing the distribution of data for the three behavioral traits analyzed in this study.


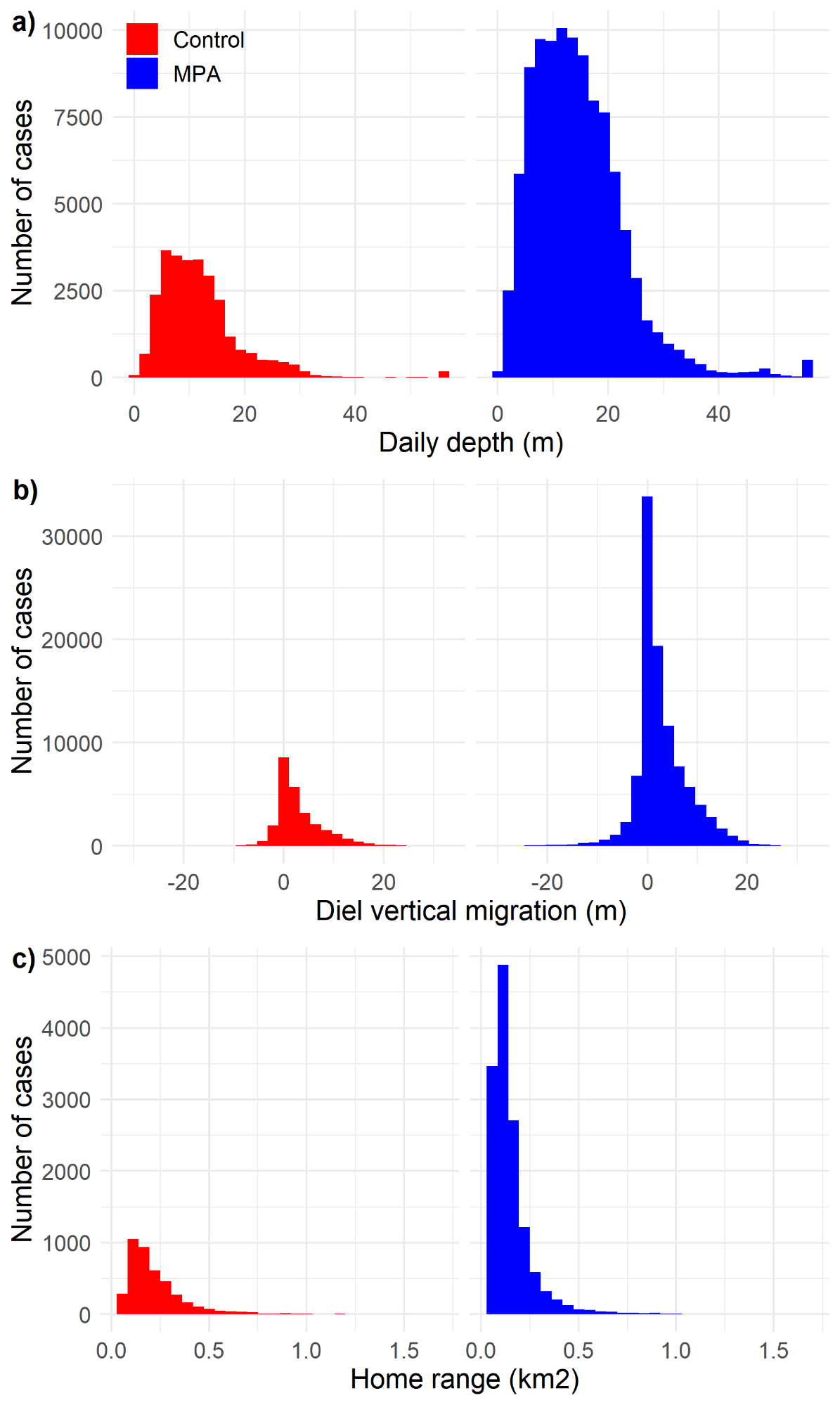


**Supplementary Figure 4.** Effect of time of the year (a, c, d) and body size (b, d e) on different behavioral traits analyzed in this study.


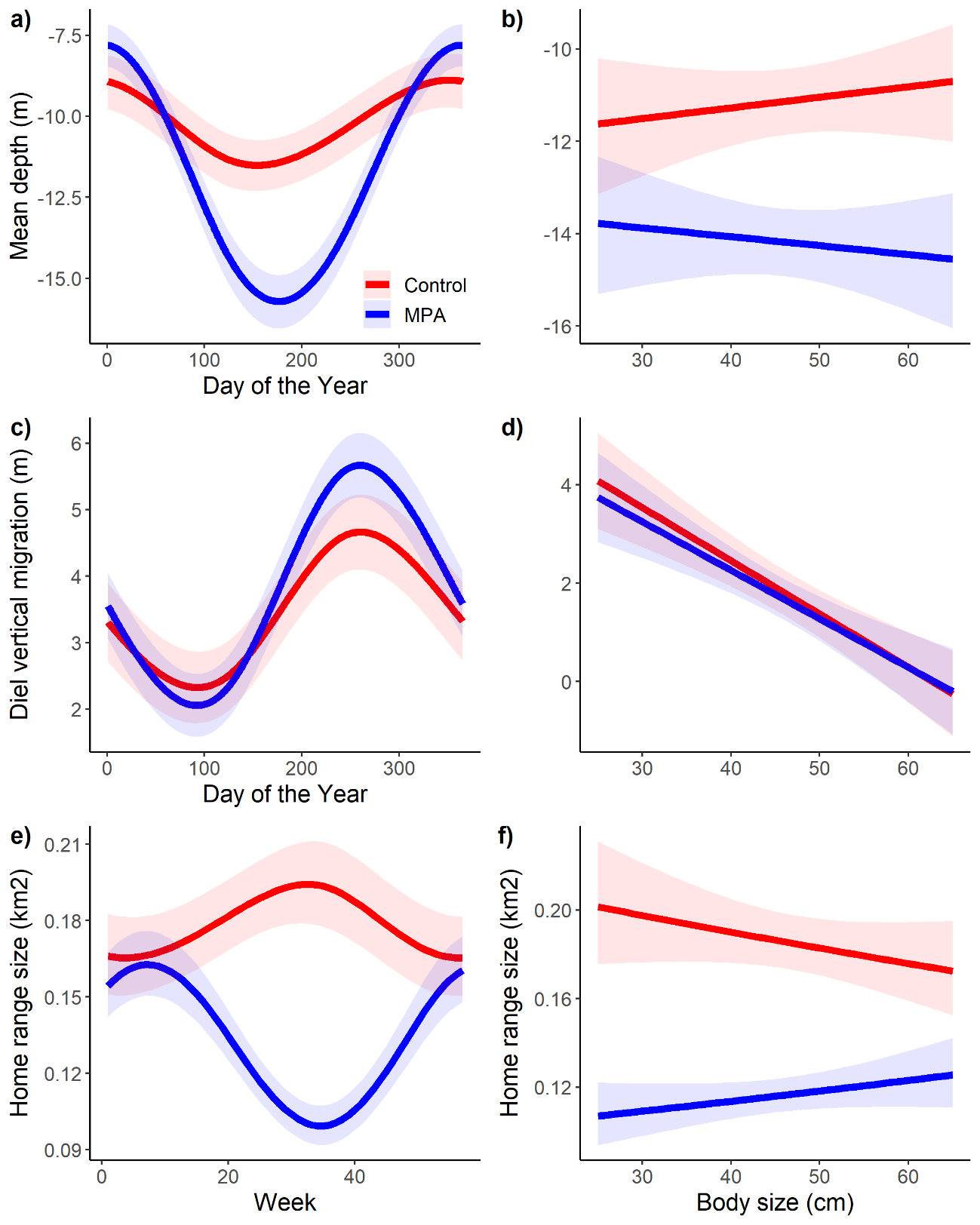

Supplement: Supplementary file 1 — Table S1 Table S2 Figure S1 Figure S2 Figure S3 Figure S4 [file EVA-15-1846-s001.docx]
